# Melt-mixed superlayer cocrystal formation using symmetric and unsymmetric organic semiconductors

**DOI:** 10.1126/sciadv.adv1878

**Published:** 2025-04-04

**Authors:** Kiyoshi Nikaido, Seita Kuroda, Satoru Inoue, Tatsuo Hasegawa

**Affiliations:** Department of Applied Physics, The University of Tokyo, Tokyo 113-8656, Japan.

## Abstract

Organic molecules with a rigid, π-conjugated core (π-core) and flexible alkyl chains (C*_n_*) naturally exhibit liquid crystal (LC) phases, promoting self-assembly of quasi–two-dimensional semiconducting layered crystals. However, particular roles of rigid and flexible parts in layer formations remain elusive. Here, we demonstrate formation of an unprecedented superlayer cocrystal phase via a unique smectic LC phase in the equimolar melt mixture of symmetrically distinct molecules. The molecules used are a monoalkylated [(π-core)-C*_n_*] using 2-octyl[1]benzothieno[3,2-*b*][1]benzothiophene (mono-C_8_-BTBT) and a dialkylated [C*_n_*-(π-core)-C*_n_*] using 2,7-dioctyl[1]benzothieno[3,2-*b*][1]benzothiophene (di-C_8_-BTBT). Thermal analyses show that the superlayer cocrystal is exclusively induced at the equimolar mixture via melt crystallization from the LC phase. X-ray structure analysis reveals a reversible C*_n_*-(π-core)-C*_n_*···(π-core)-C*_n_* stacking arrangement in the superlayer cocrystal, where π-cores and alkyl chains form nearly independent layers. Notably, this melt crystallization allows solvent-free fabrication of semiconductive polycrystalline films for excellent thin-film transistors. These findings pave the way for tailoring a quasi–two-dimensional structure in LC materials toward molecular electronics.

## Teaser

An unprecedented superlayer cocrystal emerges via melt-mixed liquid crystal in symmetric/unsymmetric organic semiconductors.

## INTRODUCTION

Rod-like organic semiconductors (OSCs) composed of a rigid, π-conjugated core (π-core) attached to flexible alkyl chains (C*_n_*) often exhibit enhanced layered crystallinity. This layered structure facilitates two-dimensional charge transport in organic thin-film transistors (OTFTs), leading to high carrier mobility, as exemplified in derivatives of benzothienobenzothiophene (BTBT) (*1*–*6*). Quantum chemical studies have attributed this high crystallinity to the synergistic interactions between the π-core and alkyl chains, both of which inherently promote layered ordering (*7*, *8*). The alkyl chains not only improve the solubility of OSCs but also stabilize the layered crystal structure, allowing for the fabrication of high-quality crystalline thin films via solution-based processes (*4*, *9*–*14*).

A fascinating property of these materials is the strong correlation between their ability to form layered crystal structures and the emergence of smectic or layered liquid crystal (LC) phases. These LC phases arise from the conformational flexibility of the alkyl chains at elevated temperatures (*15*–*18*). Recent studies suggest that the presence of an LC phase enhances the formation of layered crystalline films during solution processing of OSCs (*18*, *19*). Molecular dynamics simulations have indicated that a smectic LC–like state temporarily forms at the air-liquid interface before crystallization, acting as a precursor to the growth of crystalline thin films (*19*). During these crystallization processes, the molecules initially adopt a layered LC order, allowing their diffused motion within the molecular layers (*18*–*22*). Subsequently, long-range positional and orientational order emerges, driven by molecular motion within the LC phase (*18*, *19*). This LC-mediated crystallization differs fundamentally from conventional crystallization based on nucleation and growth, underscoring the pivotal role of the LC phase in the development of crystalline OSC films (*18*, *19*).

Moreover, LC-mediated crystallization can promote the formation of cocrystal phases in mixtures of alkylated OSCs with varying alkyl chain lengths (*18*, *23*, *24*). The LC-like state facilitates molecular diffusion within the layers, leading to complete mixing in the layered cocrystal phase. We hypothesize that such LC-phase crystallization could provide a notable approach to control the layered crystal structures of alkylated OSCs through molecular mixing. However, this mixing has typically been limited to OSCs that are structurally similar, differing primarily in their alkyl chain lengths. In liquid crystalline materials, mixing distinct molecules is a common strategy to hinder crystallization and stabilize the LC phase (*20*, *25*–*28*). This approach has also proven effective in exploring mixing-induced LC phases absent in the pure compounds (*26*–*31*). We propose that mixing OSC molecules with distinct molecular structures could offer opportunities for controlling supramolecular structures via LC-mediated crystallization.

**Fig. 1.**
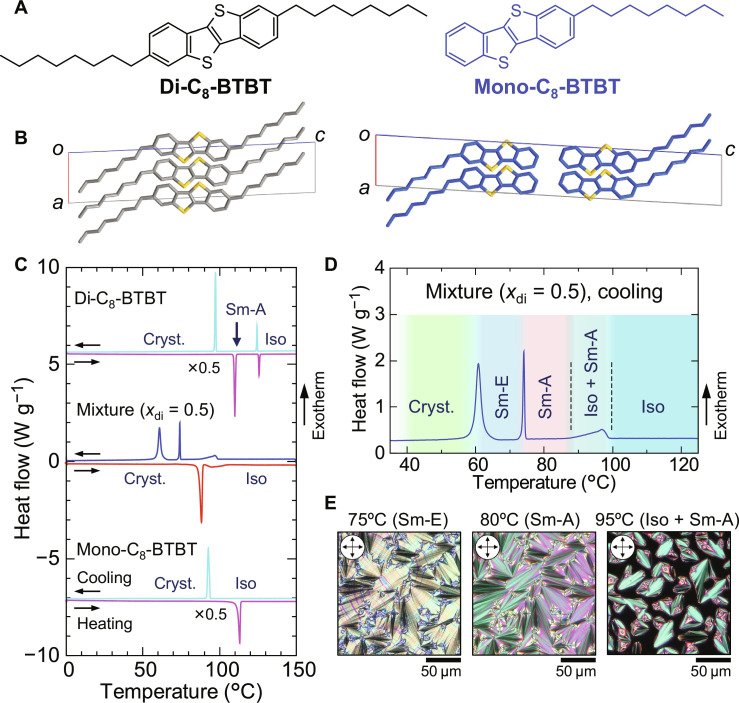
Phase transition behaviors of the equimolar mixture of di-C_8_-BTBT and mono-C_8_-BTBT. (**A** and **B**) Molecular structures and corresponding crystal structures of di-C_8_-BTBT and mono-C_8_-BTBT. The crystal structures are reproduced from crystallographic data in references (*32*, *36*). (**C**) DSC thermograms for the pristine compounds and the equimolar mixture (*x*_di_ = 0.5). The temperature scan rate is set to 2°C min^−1^, with upward peaks indicating exothermic transitions. For clarity, the DSC profiles of di-C_8_-BTBT and mono-C_8_-BTBT are scaled by a factor of 0.5 and plotted with an offset. (**D**) Magnified view of the cooling scan for the equimolar mixture, highlighting the phase transitions between 60° and 100°C. (**E**) POM images of the equimolar mixture (*x*_di_ = 0.5) during cooling, showing the phase transition sequence. At 95°C, the coexistence of Iso and Sm-A phases is observed. At 80°C, a fan-shaped texture characteristic of the Sm-A phase is visible, while at 75°C, a striated fan-shaped texture indicates the formation of the Sm-E phase. All POM images were taken after 1 min of equilibration at the respective temperatures.

In this study, we explore the effect of mixing OSC molecules with different molecular symmetries on liquid crystallinity and layered crystal structures. Specifically, we focus on the prototypical OSCs 2,7-dioctyl[1]benzothieno[3,2-*b*][1]benzothiophene (di-C_8_-BTBT) (*2*, *3*, *32*) and 2-octyl[1]benzothieno[3,2-*b*][1]benzothiophene (mono-C_8_-BTBT) (*33*–*35*) (Fig. 1, A and B), which serve as model systems. These molecules feature symmetric and unsymmetric alkyl substitutions on the BTBT π-core, forming C_8_-(π-core)-C_8_ and (π-core)-C_8_ structures, respectively. Thermodynamic analysis reveals that a smectic-E (Sm-E) LC phase is uniquely induced at elevated temperatures in the equimolar melt-mixed compound. Furthermore, powder x-ray diffraction (XRD) analysis suggests that the equimolar mixture forms a unique cocrystal phase, in which the molecules are fully mixed, leading to a distinctive multilayer superstructure composed of a reversible C_8_-(π-core)-C_8_···(π-core)-C_8_ stacking configuration. To understand the mechanisms behind these mixing-induced phases, we investigate the roles of alkyl chains and π-cores in stabilizing both LC and cocrystal phases using phase diagrams, structural characterization, and crystallization kinetics. We further demonstrate that crystallization from the Sm-E phase in the equimolar mixture enables the solvent-free fabrication of crystalline films for OTFTs through melt crystallization.

## RESULTS

### Emergence of a melt-induced superlayer phase at the equimolar mixture

The thermal properties of pristine mono-C_8_-BTBT, di-C_8_-BTBT, and their equimolar mixture (*x*_di_ = 0.5, where *x*_di_ represents the mole fraction of di-C_8_-BTBT in the mixture) were investigated using differential scanning calorimetry (DSC). As previously reported (*8*, *34*), di-C_8_-BTBT undergoes a phase transition to a smectic-A (Sm-A) phase, while mono-C_8_-BTBT exhibits only a single phase transition at its melting point without any evidence of LC behavior (Fig. 1C). In contrast, the equimolar mixture (*x*_di_ = 0.5) displays three distinct exothermic peaks during the cooling scan: a broad peak between 90° and 100°C, followed by the onset of sharp peaks at 75.0° and 66.5°C (Fig. 1D provides a magnified view). Notably, the phase transition behavior is unaffected by the cooling rate, as demonstrated by consistent phase sequences and latent heat measurements across different cooling rates (fig. S1). These observations suggest the formation of a previously unobserved phase in the equimolar mixture, absent in the pristine compounds.

To better understand the high-temperature phases detected by DSC, we examined the optical textures of the equimolar mixture by polarized optical microscopy (POM) within a custom-built LC cell. We observed the coexistence of isotropic liquid (Iso) and Sm-A phases between 90° and 100°C, fan-shaped Sm-A textures at 80°C, and striated fan-shaped Sm-E textures at 75°C (Fig. 1E and fig. S2). This LC phase transition was not detected during the heating scan (Fig. 1C), indicating that the mixing-induced Sm-E phase is monotropic, emerging only upon cooling from the melt.

**Fig. 2.**
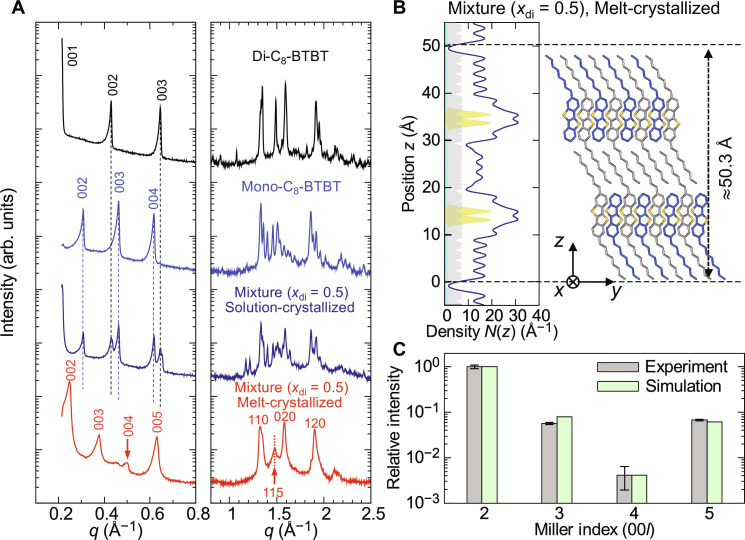
Structural characterization of the equimolar mixture of di-C_8_-BTBT and mono-C_8_-BTBT in the crystal phase. (**A**) Powder XRD profiles of the pristine compounds (di-C_8_-BTBT and mono-C_8_-BTBT), the equimolar mixture crystallized from solution, and the melt-crystallized equimolar mixture. The as-grown, solution-crystallized sample shows phase separation between the two components, whereas the melt-crystallized mixture exhibits a distinct cocrystal phase without phase separation. (**B**) Electron density profiles along the *z* axis (left) and a schematic of the layered crystal structure in the melt-crystallized equimolar mixture (right). The shaded areas in the electron density profile represent the contributions of carbon (gray), hydrogen (cyan), and sulfur (yellow), while the solid line represents the total electron density. The schematic illustrates the alternating stacking of di-C_8_-BTBT and mono-C_8_-BTBT molecules, forming a superlayer structure. (**C**) Comparison of the normalized diffraction intensities of the 00*l* Bragg peaks, derived from experimental data and simulations. Error bars in the experimentally determined intensities represent the uncertainty in the Lorentzian fitting of the spectra.

We further investigated the room-temperature crystal structure of each compound using powder XRD (Fig. 2A). The XRD patterns of the pristine compounds were consistent with previously reported crystal structures (*8*, *32*, *35*, *36*): Di-C_8_-BTBT adopts a layered herringbone (LHB) structure, while mono-C_8_-BTBT forms a bilayer-type layered herringbone (b-LHB) structure, characterized by core-to-core and chain-to-chain interlayer stacking [C_8_-(π-core)··(π-core)-C_8_] (Fig. 1B). Notably, solution-crystallized equimolar mixtures exhibited phase separation, with distinct LHB and b-LHB phases corresponding to di-C_8_-BTBT and mono-C_8_-BTBT, respectively, as evidenced by the superimposed XRD profiles of the parent compounds (left panel, Fig. 2A).

However, when the equimolar mixture was subjected to melt crystallization—by heating to 150°C and then cooling to room temperature—the XRD pattern showed no peaks corresponding to the parent compounds (Fig. 2A). This finding clearly indicates the formation of a cocrystal phase in the melt-mixed equimolar mixture without phase separation. The XRD pattern could be indexed assuming an orthorhombic lattice, with lattice constants of *a* ≈ 5.92 Å, *b* ≈ 7.95 Å, and *c* ≈ 50.3 Å. The observed Bragg peaks at 110, 020, and 120 (right panel, Fig. 2A) suggest an in-plane herringbone arrangement of the π-cores, and the lattice constants *a* and *b* are comparable to those of the parent compounds (*8*, *32*, *35*, *36*), indicating that the herringbone π-core arrangement is preserved in the melt-crystallized mixture.

The layer spacing of *c* = 50.3 Å corresponds approximately to the combined molecular lengths of di-C_8_-BTBT (~3 nm) and mono-C_8_-BTBT (~2 nm). We propose that this observed layer spacing arises from a cocrystal structure in which mono-C_8_-BTBT and di-C_8_-BTBT molecules are alternately stacked. In this structure, the alkyl chains and π-cores form distinct layers, following a reversible C_8_-(π-core)-C_8_···(π-core)-C_8_ stacking motif (Fig. 2B). To validate this proposed structure, we simulated the relative intensities of the 00*l* Bragg peaks on the basis of the electron density profile (left panel, Fig. 2B) and compared the calculated diffraction intensities with the experimental data. The electron density along the layer normal (*z* axis) was approximated as a superposition of Gaussian distributions

$$N\left(z\right)=\sum_{j}Z_{j}\text{exp}\left[-\frac{{\left(z-z_{j}\right)}^{2}}{2\sigma^{2}}\right]$$ (1)

where *Z_j_* and *z_j_* represent the number of electrons and *z*-coordinates of the *j*-th atom, and σ is the degree of Gaussian disorder of the electron density (*37*). We calculated the structure factor for indexing 00*l* using

$$F_{00l}=\int_{0}^{d_{001}}N\left(z\right)\text{exp}\left(2\pi i\frac{\mathrm{lz}}{d_{001}}\right)\mathrm{dz}$$ (2)

The diffraction intensity was then calculated using *I*_00*l*_ ∝ |*F*_00*l*_|^2^*L*(θ_00*l*_), where θ_00*l*_ is the diffraction angle of the 00*l* Bragg peak, and *L*(θ) = (4 sin^2^θ cosθ)^−1^ is the Lorentz factor from the Debye-Scherrer method (*38*). In this simulation, we optimized the Gaussian disorder σ in Eq. 1 and the interlayer distance between BTBT cores within the unit cell to obtain the best fit to the observed 00*l* intensity (see Supplementary Text and fig. S3 for details of parameter optimization). As shown in Fig. 2C, the simulated Bragg peak intensities align well with the experimental data, particularly the substantially weaker intensity of the 004 peak.

These results confirm that the equimolar mixture forms a unique cocrystal phase upon melt crystallization, where alkyl chains and π-cores are independently aligned within the layers, as schematically illustrated in Fig. 2B and fig. S4. Given the strong layer-forming ability of both the alkyl-chain (C_8_) layers and π-core layers, it is likely that the stacking of the distinct molecules involves orientational disorder in their long-axis alignment. We suggest that the ordering ability of both π-core and alkyl-chain layers takes precedence over phase separation, ultimately leading to the formation of a unique “superlayer” cocrystal phase that extends beyond the typical interlayer period.

### Structure of the melt-induced liquid-crystal phase in equimolar mixtures

**Fig. 3.**
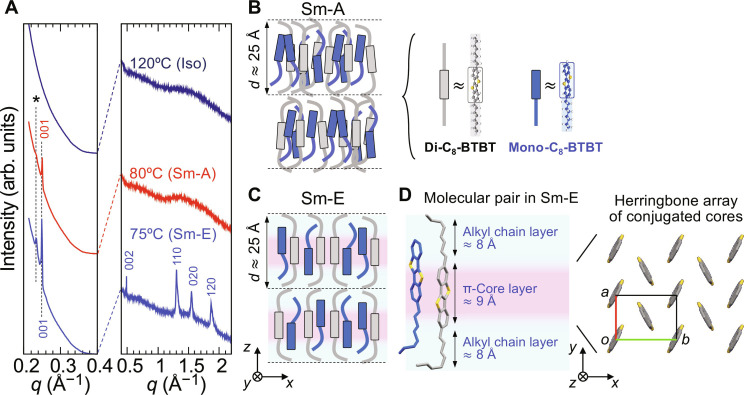
Structure of the LC phase in the equimolar mixture of di-C_8_-BTBT and mono-C_8_-BTBT. (**A**) Temperature-controlled powder XRD profiles of the equimolar mixture, showing the evolution of Bragg peaks with temperature. The 001 Bragg peaks indicate layer spacings of *d*_001_ ≈ 25.2 Å in the Sm-E phase and *d*_001_ ≈ 25.1 Å in the Sm-A phase. An additional peak (denoted by an asterisk) corresponds to a slightly larger layer spacing of ≈ 26.6 Å. (**B**) Schematic representation of the molecular arrangement in the Sm-A phase, where the molecules are uniformly mixed, and the layer spacing is determined by the total volume of the alkyl chains. (**C**) Schematic for the molecular arrangement in the Sm-E phase, where the emergence of in-plane order leads to three-dimensional periodicity. (**D**) (Left) Hypothetical model showing alternating pairs of di-C_8_-BTBT and mono-C_8_-BTBT molecules in the Sm-E phase, where the π-cores are densely packed, and the alkyl chains are conformationally disordered. (Right) Schematic of the in-plane herringbone packing of the π-cores in the Sm-E phase.

To elucidate the structure of the LC phases in the equimolar mixture, we performed temperature-controlled powder XRD measurements (Fig. 3A), which corroborated the POM observations shown in Fig. 1E. At 80°C, we observed only the 001 Bragg peak, indicating the presence of layered order without in-plane molecular arrangement, characteristic of the Sm-A phase. As the temperature was lowered to 75°C, additional in-plane *hk*0 Bragg peaks emerged alongside the 001 Bragg peak, revealing the onset of three-dimensional periodicity typical of the Sm-E phase. On the basis of the XRD profile (Fig. 3A), we determined the layer spacing in the Sm-A and Sm-E phases to be ~25.1 and 25.2 Å, respectively, with a minor fraction showing a slightly larger layer spacing of 26.6 Å (denoted by an asterisk in Fig. 3A). It is noteworthy that the layer spacings exhibit slight temperature dependence (fig. S5).

To gain further insight into the molecular arrangement in the smectic LC phases, we investigated the layer spacing of the Sm-A phase at various compositions. Mixtures with *x*_di_ ≳ 0.3 exhibit Sm-A phases at elevated temperatures. The layer spacings in the Sm-A phase increase monotonically with *x*_di_ from ~25 Å at *x*_di_ = 0.5 to around 29 Å at *x*_di_ = 0.8 (fig. S5). This trend suggests that di-C_8_-BTBT and mono-C_8_-BTBT molecules are uniformly mixed within the molecular layers, as illustrated in Fig. 3B. The layer spacing appears to be determined primarily by the total volume of the alkyl chains, which increases with the addition of di-C_8_-BTBT. Notably, the observed layer spacings are longer than the molecular length of mono-C_8_-BTBT (~2 nm) but shorter than that of di-C_8_-BTBT (~3 nm), indicating that the alkyl chains are conformationally disordered rather than adopting a fully extended all-trans configuration.

In contrast, the Sm-E phase at *x*_di_ = 0.5 exhibits distinct in-plane periodicity, as evidenced by the 110, 020, and 120 Bragg peaks (Fig. 3A), which likely reflect a herringbone arrangement of the π-cores within the molecular layers. The in-plane lattice constants (*a* ≈ 5.98 Å and *b* ≈ 8.13 Å) are comparable to those of the crystalline phase, suggesting dense π-core packing in a herringbone arrangement. Previous studies have shown that alkyl chains in the Sm-E phase are disordered, while the π-cores remain closely packed (*15*–*18*, *39*, *40*). As for the present case, the conformational disorder of alkyl chains in the Sm-E phase is associated with the large entropy change (~60 J K^−1^ mol^−1^) along with the phase transition from the Sm-E phase to the crystal phase as shown in fig. S1C. On the basis of these findings, we propose that di-C_8_-BTBT and mono-C_8_-BTBT molecules alternate along parallel to the layer in the Sm-E phase, as schematically illustrated in Fig. 3C. The disordered alkyl chains of di-C_8_-BTBT likely occupy the space above and below adjacent mono-C_8_-BTBT molecules (Fig. 3D), allowing the π-cores to pack tightly. This model indicates that the 1:1 pairing of di-C_8_-BTBT and mono-C_8_-BTBT is essential for inducing the Sm-E phase, and equimolar mixing is likely required to stabilize it.

### Temperature-concentration diagram for melt-induced phases

**Fig. 4.**
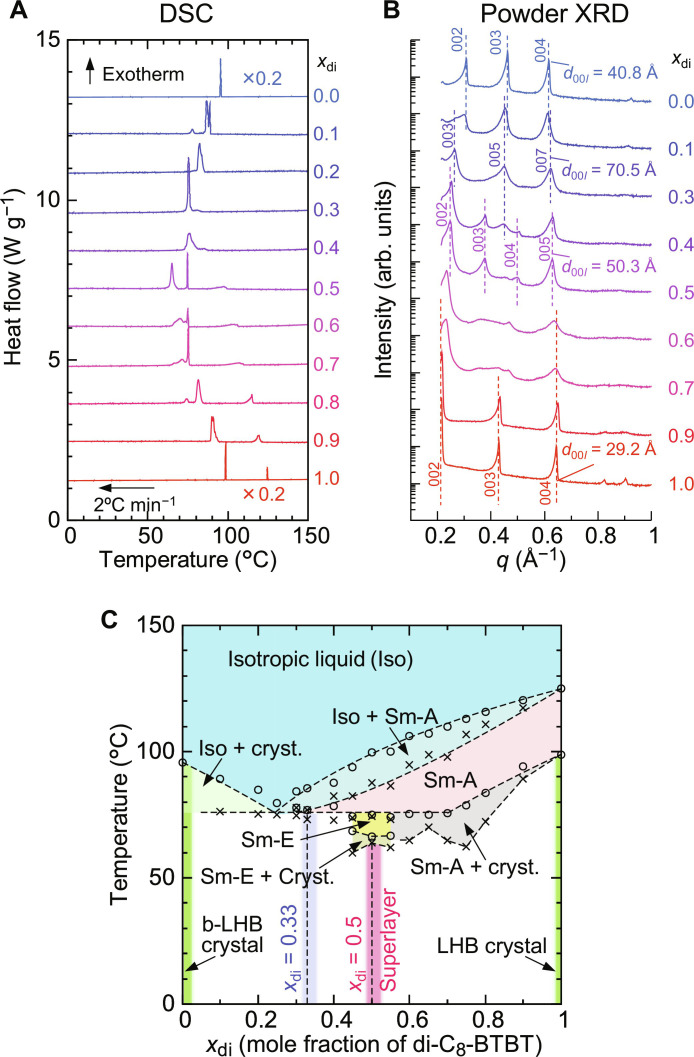
Thermal properties of the mixtures of mono-C_8_-BTBT and di-C_8_-BTBT. (**A**) DSC thermograms during the cooling scan, with *x*_di_ representing the mole fraction of di-C_8_-BTBT in the mixtures. The cooling rate is set to 2°C min^−1^. To enhance clarity, the DSC profiles for the pristine compounds (*x*_di_ = 0.0 and 1.0) are scaled by a factor of 0.2 and plotted with offsets. Solution-crystallized samples were used for the pristine compounds, while melt-crystallized samples were used for the mixtures. (**B**) Powder XRD profiles of the mixtures measured at room temperature, revealing the structural variations across different compositions. (**C**) Temperature-concentration phase diagram of the mixtures. Open circles and crosses denote the onset and offset temperatures of phase transitions, as determined by DSC and POM observations. Dashed lines indicate empirical fits to the phase boundaries. Pure crystalline phases were observed at *x*_di_ ~ 0.0, 0.33, 0.5, and 1.0, which are colored with green, blue, red, and green lines, respectively, while the white regions represent the mixed crystalline states composed of the several crystal forms.

To explore the phase behavior of mixtures with varying compositions (*x*_di_), we constructed a temperature-concentration phase diagram on the basis of DSC and powder XRD measurements. The DSC scans (Fig. 4A and fig. S5) reveal that the number of phase transitions and transition temperatures is highly dependent on the mixture composition. Powder XRD analysis of the melt-crystallized mixtures at room temperature (Fig. 4B and fig. S5) shows distinct structural trends: Mixtures with *x*_di_ = 0.6 and 0.7 exhibit much broader diffraction patterns than those with other compositions, indicating the coexistence of pristine di-C_8_-BTBT crystals and a superlayer cocrystal phase (*x*_di_ = 0.5). In contrast, the mixtures with *x*_di_ = 0.3 exhibit a larger layer spacing of 70.5 Å, as evidenced by the 003, 005, and 007 Bragg peaks (Fig. 4B). Although the crystal structure at *x*_di_ = 0.3 is not fully characterized, we speculate that it represents a 2:1 mixture of mono-C_8_-BTBT and di-C_8_-BTBT (*x*_di_ ≈ 0.33), as the layer spacing of 70.5 Å corresponds to the combined molecular lengths of two mono-C_8_-BTBT molecules and one di-C_8_-BTBT molecule, likely forming another superlayer cocrystal phase. Mixtures with *x*_di_ = 0.1 and *x*_di_ = 0.4 display Bragg peaks corresponding to *x*_di_ = 0.3 and 0.5, indicating the coexistence of multiple crystal phases.

On the basis of DSC cooling scans, powder XRD, and POM observations (figs. S6 to S8), we identified each LC phase and constructed the temperature-concentration phase diagram shown in Fig. 4C. The phase transition temperatures were determined by DSC, with the onset and offset temperatures of the exothermic peaks plotted as open circles and crosses, respectively, in Fig. 4C. Compared to the pure compounds, the mixtures exhibit broader exothermic peaks during cooling, corresponding to two-phase coexistence during the transitions from the Iso phase to the Sm-A, crystal, and Sm-E phases. The broad temperature range of two-phase coexistence, as shown in the phase diagram, reflects the two degrees of freedom for two-phase equilibrium in binary mixtures according to Gibbs’ phase rule.

The Sm-A phase appears only in mixtures with *x*_di_ ≳ 0.3, while no LC phases are observed for *x*_di_ ≲ 0.3. The mixing-induced Sm-E phase is exclusive to the vicinity of equimolar composition (*x*_di_ = 0.5). This selective stabilization of the Sm-E phase near *x*_di_ = 0.5 is consistent with the structure proposed in the previous section, where the 1:1 pairing of di-C_8_-BTBT and mono-C_8_-BTBT is crucial for stabilizing the Sm-E phase. As the fraction of di-C_8_-BTBT increases (*x*_di_ > 0.5), the excess disordered alkyl chains from di-C_8_-BTBT disrupt the close packing of the π-cores in a herringbone arrangement. Conversely, when the fraction of mono-C_8_-BTBT increases (*x*_di_ < 0.5), the reduced number of alkyl chains leads to insufficient packing in the LC phases, weakening the intermolecular interactions and destabilizing the Sm-E phase. Thus, the Sm-E phase is uniquely stabilized at the equimolar composition.

### Isothermal melt-crystallization kinetics for superlayer formation

**Fig. 5.**
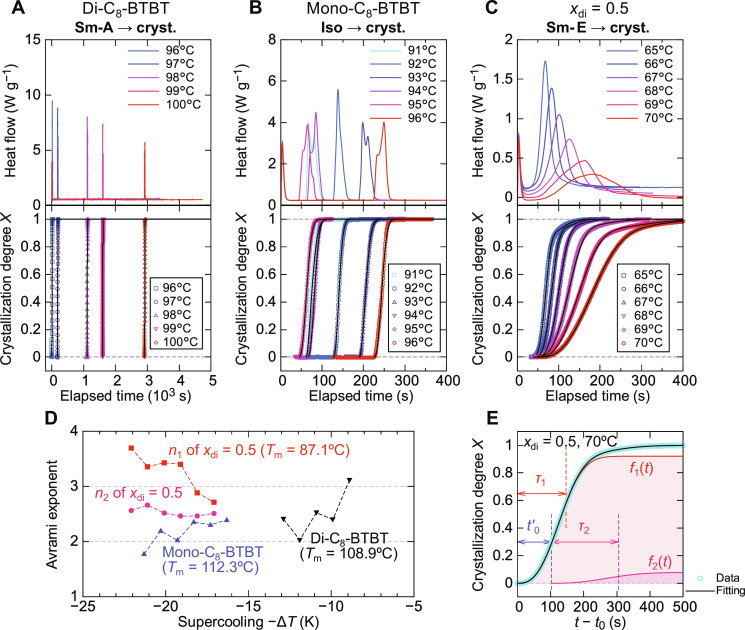
Isothermal crystallization kinetics. (**A** to **C**) DSC thermograms under isothermal conditions (upper panels) and the time evolution of the crystallization degree *X* (lower panels) for crystallization from the Sm-A, Iso, and Sm-E phases in di-C_8_-BTBT, mono-C_8_-BTBT, and the equimolar mixture (*x*_C8_ = 0.5), respectively. The black solid lines in the lower panels represent the fits to the KJMA model using Eq. 4 for di-C_8_-BTBT and mono-C_8_-BTBT and Eq. 5 for *x*_di_ = 0.5. (**D**) Temperature dependence of the Avrami exponents, plotted as a function of the supercooling degree −Δ*T* = −(*T*_m_ − *T*). Squares and circles denote the Avrami exponents *n*_1_ and *n*_2_ for *x*_di_ = 0.5, while upward and downward triangles represent the Avrami exponents of mono-C_8_-BTBT and di-C_8_-BTBT, respectively. (**E**) Fitting of the crystallization kinetics for *x*_di_ = 0.5 at 70°C, showing the distinct crystallization stages as described by the two-step process.

In this section, we explore the isothermal crystallization kinetics of the pristine compounds and the equimolar mixture (*x*_di_ = 0.5) of mono-C_8_-BTBT and di-C_8_-BTBT under supercooling conditions using heat compensation DSC. To assess the time evolution of crystallization, we first melted each sample at 150°C and then rapidly cooled it to the crystallization temperature at rates of 20°C min^−1^ for *x*_di_ = 0.5 and 60°C min^−1^ for the pristine compounds. During isothermal crystallization, DSC traces exothermic peaks corresponding to crystallization transitions: from the Iso phase to the crystal phase in mono-C_8_-BTBT, from the Sm-A phase to the crystal phase in di-C_8_-BTBT, and from the Sm-E phase to the crystal phase in the equimolar mixture (Fig. 5, A to C, upper panels). The degree of crystallization, *X*(*t*), was estimated from the DSC measurements as follows (*41*–*43*)

$$X\left(t\right)=\frac{1}{\Delta H}\int_{t_{0}}^{t}{\left(\frac{\partial H}{\partial t}\right)}_{P}\mathrm{dt}$$ (3)

where *t*_0_ is the onset of crystallization, Δ*H* is the total enthalpy change, and (∂H/∂t)*_P_* is the DSC signal. As shown in the lower panels of Fig. 5 (A to C), crystallization begins after a short incubation time and is completed within 10 to 300 s.

For both di-C_8_-BTBT and mono-C_8_-BTBT (Fig. 5, A and B), the crystallization kinetics can be described by the Kolmogorov-Johnson-Mehl-Avrami (KJMA) model (*43*–*46*)

$$X\left(t\right)=1-\text{exp}\left[-{\left(\frac{t-t_{0}}{\tau_{\text{cr}}}\right)}^{n}\right]$$ (4)

where *n* is the Avrami exponent, *t*_0_ is the incubation time, and τ_cr_ is the characteristic crystallization time (fitting curves and parameters are provided in figs. S9 and S10 and tables S1 to S3). The crystallization time tends to decrease as the temperature decreases, as shown by the time-temperature-transformation diagram in fig. S11, indicating that crystallization is driven primarily by the thermodynamic nucleation and growth mechanism rather than by thermally activated molecular diffusion (*41*, *42*, *47*, *48*).

The Avrami exponents for the pristine compounds, plotted against the degree of supercooling Δ*T* = (*T*_m_ – *T*) (where *T*_m_ is the melting temperature), typically range between 2 and 3 (Fig. 5D). This suggests that crystallization in di-C_8_-BTBT and mono-C_8_-BTBT proceeds via two-dimensional crystal growth (*45*), consistent with the formation of thin flake crystals (*8*).

In contrast, the crystallization kinetics of the equimolar mixture (*x*_di_ = 0.5) cannot be described by a single KJMA function. Instead, the crystallization process appears to involve two distinct stages, as described in (*42*)

X(t)={f1(t), for t0≤t<t0+t0′f1(t)+f2(t), for t≥t0+t0′ (5)

where $f_{1}\left(t\right)$ and $f_{2}\left(t\right)$ represent the first and second crystallization processes, respectively, with $t_{0}^{\prime }$ being the delay time before the second process. The two stages are described by

$$f_{1}\left(t\right)=A\left\{1-\text{exp}\left[-{\left(\frac{t-t_{0}}{\tau_{1}}\right)}^{n_{1}}\right]\right\}$$ (6)

$$f_{2}\left(t\right)=\left(1-A\right)\left\{1-\text{exp}\left[-{\left(\frac{t-t_{0}-t_{0}^{\prime }}{\tau_{2}}\right)}^{n_{2}}\right]\right\}$$ (7)

where *A* is the fraction of the first crystallization process, and *n*_1_, *n*_2_, τ_1_, and τ_2_ represent the Avrami exponents and characteristic crystallization times for the two processes. Fitting the data using this two-stage model (Fig. 5, C and E) indicates that the crystallization from the Sm-E phase to the superlayer cocrystal phase in the equimolar mixture involves two parallel processes.

Both τ_1_ and τ_2_ show similar temperature dependence (fig. S11 and table S1), with crystallization times decreasing as the temperature decreases, confirming that crystallization is driven thermodynamically. However, the total crystallization time (100 to 300 s) is an order of magnitude longer than for the pristine compounds, likely due to the additional time required for the formation of molecular pairs between di-C_8_-BTBT and mono-C_8_-BTBT in the superlayer phase.

Further analysis of the Avrami exponents for the two stages of crystallization (Fig. 5D) shows that the first crystallization process exhibits exponents between 2.7 and 3.7, suggesting two- to three-dimensional growth (*45*). We propose that this stage involves the stacking of molecular layers to establish long-range interlayer order with a periodicity of 50.3 Å in the superlayer cocrystal phase. As the molecular layers are already partially ordered in the Sm-E phase, this initial crystallization is likely driven by reorientation of mono-C_8_-BTBT molecules and the diffusion and pairing of di-C_8_-BTBT and mono-C_8_-BTBT, leading to the superlayer structure (Fig. 2B). This process proceeds in three dimensions, as indicated by the higher Avrami exponent (*n*_1_ > 3).

In contrast, the second crystallization stage, with an Avrami exponent of ~2.5, involves two-dimensional growth (*45*). This stage represents the completion of long-range order in the superlayer cocrystal phase, confined within the molecular layers (fig. S12). We suggest that the grain structure of the superlayer cocrystal phase is inherited from the Sm-E phase, which serves as a precursor to superlayer formation.

### Thin-film formation via melt-mixed superlayers

**Fig. 6.**
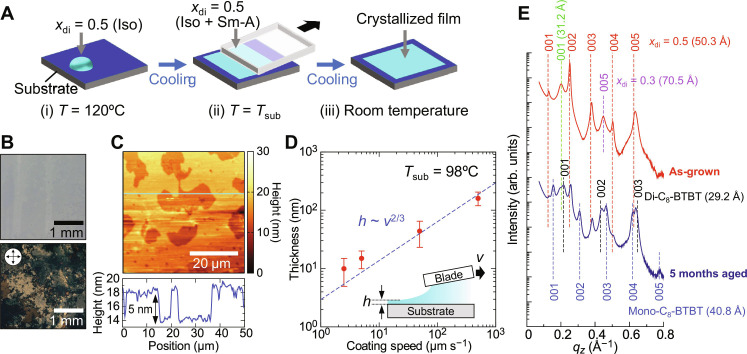
Solvent-free film coating process for the equimolar mixture of mono-C_8_-BTBT and di-C_8_-BTBT. (**A**) Schematic of the solvent-free blade-coating process. The substrate temperature during the coating process was set between 95° and 105°C. (**B**) Optical microscopy image of the crystalline film obtained with a coating speed of 5 μm s^−1^. The lower panel shows a POM image of the film. (**C**) AFM topography of the film surface. The lower panel displays a cross-sectional height profile along the horizontal line (cyan) in the AFM image. (**D**) Relationship between the film thickness (*h*) and coating speed (*v*) at *T*_sub_ = 98°C. (**E**) Out-of-plane XRD profiles of the crystalline films for both the as-grown (1 day aged) and 5-month-aged samples.

In this section, we demonstrate that the emergence of the highly ordered Sm-E liquid-crystalline phase enables the solvent-free fabrication of polycrystalline films, combining blade-coating techniques with a melt-crystallization process. Figure 6A illustrates the solvent-free coating process for the equimolar mixture, which involves the following steps: (i) The compounds are melted by heating the substrate; (ii) the substrate is cooled to the coating temperature (*T*_sub_), and the molten droplet is spread across the surface via mechanical shear using a sweeping blade (an untreated glass plate); and (iii) the substrate is then cooled to room temperature at a controlled rate of ~2°C min^−1^. In this process, the compounds are initially melted by heating the substrate to 120°C, and the coating temperature is set to a point where the Iso and Sm-A phases coexist.

A silicon wafer with a 100-nm-thick thermally grown SiO_2_ layer was used as the substrate to coat the equimolar mixture. Figure 6B shows an optical microscopy image of the resulting film, produced at *T*_sub_ = 98°C and a coating speed of *v* = 5 μm s^−1^. Although some pinholes are visible (fig. S13), the film is largely continuous across a centimeter-scale area. The lower panel of Fig. 6B and fig. S14 presents a POM image, revealing grains with varying orientations, as evidenced by differences in brightness, confirming the film’s polycrystalline nature. Atomic force microscopy (AFM) topography (Fig. 6C) further highlights the step and terrace structure of the film surface, with step heights of ~5 nm, consistent with the layer spacing of the crystal phase in the equimolar mixture (*x*_di_ = 0.5).

For comparison, we also fabricated crystalline films of pristine di-C_8_-BTBT using the same solvent-free process, which only exhibits an Sm-A phase. The coating temperature was set to *T*_sub_ = 131°C, near the Iso-to-Sm-A phase transition, and a coating speed of 5 μm s^−1^ was used. While the film was successfully deposited on the substrate, it exhibited periodic cracks (fig. S13), likely caused by volume contraction during crystallization from the Sm-A phase (*49*). In contrast, the Sm-E phase in the equimolar mixture minimizes abrupt volume changes during crystallization, resulting in more continuous films.

We then examined the relationship between coating speed and film thickness for the equimolar mixture. As shown in Fig. 6D, the film thickness (*h*) increases monotonically with coating speed (*v*). The thickness follows a scaling relation of *h* ~ *v*^2/3^ (Fig. 6D), which is consistent with the Landau-Levich-Derjaguin model (*50*–*52*). According to this model, higher coating speeds lead to increased film thickness, as the viscous force drags more material onto the substrate (*50*). This suggests that the Sm-A membrane is directly deposited onto the substrate, followed by crystallization upon cooling.

We also investigated how coating temperature affects the film thickness. With the coating speed fixed at *v* = 50 μm s^−1^ and *T*_sub_ varied between 93° and 108°C, the film thickness decreased as the coating temperature increased (fig. S15). This behavior likely arises because the shear modulus of the molten OSC decreases with increasing temperature (*34*), reducing the viscous force and resulting in thinner films.

In this solvent-free coating process, we anticipate that the interface between the air and the surface of the Sm-A membrane should promote homeotropic alignment (*53*), leading to the formation of crystalline films with molecular layers parallel to the substrate. To verify the film structure, we performed out-of-plane thin-film XRD measurements. The XRD profile of the as-grown film (Fig. 6E) reveals that the film primarily consists of the superlayer cocrystal phase with a layer spacing of 50.3 Å, consistent with the powder XRD data. The observation of 00*l* Bragg peaks confirms that the molecular layers (i.e., {001} planes) are oriented parallel to the substrate. However, the film also contains traces of other crystalline phases, including a cocrystal phase of *x*_di_ = 0.3 (indicated by the 005 Bragg peak, with a layer spacing of ~70.5 Å) and a di-C_8_-BTBT-rich phase (with a layer spacing of ~31.2 Å, indicated by the 001 Bragg peak).

It is worth noting that the crystalline film undergoes structural changes over time. After 5 months, the XRD profile (Fig. 6E) shows that phase separation has occurred, with Bragg peaks corresponding to the pristine compounds mono-C_8_-BTBT and di-C_8_-BTBT becoming apparent. In addition, the film’s morphology degrades over time, as shown in fig. S16, with the continuous film becoming discontinuous after 5 months of aging. These results suggest that the superlayer cocrystal phase in the equimolar mixture (*x*_di_ = 0.5) is a kinetically favored metastable phase that forms upon crystallization from the Sm-E phase.

### Device characteristics of superlayer OTFTs

**Fig. 7.**
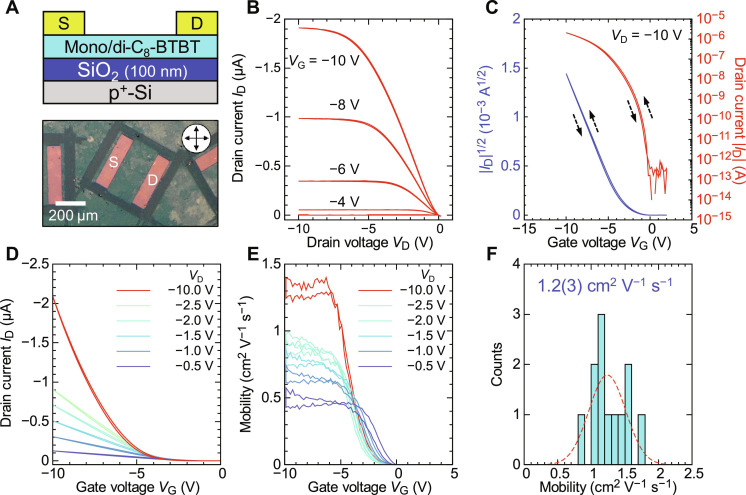
Carrier transport characteristics of solvent-free coated OTFTs using the equimolar mixture of mono-C_8_-BTBT and di-C_8_-BTBT as the active layer. (**A**) Schematic of the OTFT device geometry and a POM image of the fabricated device. (**B**) Output characteristics of the OTFT. (**C** and **D**) Transfer characteristics at different drain voltages. The channel dimensions are *L* = 200 μm and *w* = 400 μm. (**E**) Gate voltage dependence of the field-effect mobility. (**F**) Statistical distribution of the field-effect mobility in the saturation regime (*V*_D_ = −10 V), with the dotted line representing a Gaussian fit for guidance.

We fabricated OTFTs using solvent-free coated films of the equimolar mixture (*x*_di_ = 0.5) as the active layer and characterized their carrier transport properties. After depositing gold (Au) electrodes onto the as-grown films to form the source and drain contacts (Fig. 7A), the as-fabricated OTFTs initially displayed a high threshold voltage (*V*_th_ ~ −33 V, fig. S17). However, after ~2 weeks of aging, the threshold voltage substantially decreased to *V*_th_ ~ −3.2 V, as shown in the output and transfer characteristics in Fig. 7 (B and C). We speculate that the high threshold voltage in the as-fabricated OTFTs originates from structural defects in the crystalline films caused by thermal damage during electrode deposition. These defects likely generate deep trap states above the highest occupied molecular orbital level of the OSC, substantially increasing the threshold voltage (*54*). However, we hypothesize that structural relaxation over 2 weeks of aging at room temperature mitigates these defects, reducing the deep trap states and consequently lowering the threshold voltage over time.

The OTFTs exhibited hole transport characteristics with a field-effect mobility of ≳1 cm^2^ V^−1^ s^−1^ in the saturation regime (Fig. 7, D and E). The mobility in the linear regime was lower, likely due to the interlayer access resistance caused by the alkyl chain layers between the π-cores (*5*). Figure 7F shows the distribution of the field-effect mobility in the saturation regime, indicating that the solvent-free coated OTFTs consistently achieved mobilities of ~1 cm^2^ V^−1^ s^−1^. Given that the in-plane lattice constants of the superlayer cocrystal phase (*a* ≈ 5.92 Å and *b* ≈ 7.95 Å) closely match those of the pristine di-C_8_-BTBT crystal [*a* = 5.927(7) Å and *b* = 7.88(*1*) Å] (*32*), we consider that the in-plane herringbone arrangement of BTBT cores in the superlayer cocrystal phase is similar to that of pristine di-C_8_-BTBT. Consequently, the solvent-free coated polycrystalline films of the superlayer cocrystal exhibit high mobility, comparable to that of spin-coated polycrystalline di-C_8_-BTBT films (*2*). This suggests that the crystallization kinetics of the superlayer cocrystal phase, which retains the layered structure of the Sm-E phase, may suppress grain boundary formation, contributing to the stable switching characteristics observed in these OTFTs.

## DISCUSSION

We successfully demonstrated the self-organized formation of a unique superlayer cocrystal phase through the equimolar mixing of di-C_8_-BTBT and mono-C_8_-BTBT. The superlayer phase consists of multilayer structures, represented by a reversible C*_n_*-(π-core)-C*_n_*···(π-core)-C*_n_* arrangement, in which the π-cores and alkyl chains self-organize into nearly independent, alternating stacked layers. Notably, this cocrystal phase is achieved only through monotropic melt crystallization from the Sm-E phase at elevated temperatures.

Detailed DSC and XRD measurements, along with the constructed temperature-concentration phase diagram, reveal that the Sm-E phase forms exclusively at a 1:1 composition. This finding indicates that the pairing of di-C_8_-BTBT and mono-C_8_-BTBT effectively stabilizes both the Sm-E phase and superlayer cocrystal phase despite the distinct layer structures between these phases. In contrast, the Sm-A phase appears over a wider compositional range without herringbone-type intralayer order.

The isothermal crystallization kinetics further show that the transition from the mixing-induced Sm-E phase to the superlayer cocrystal phase requires an extended crystallization time compared to the pristine compounds, suggesting that the grain structure of the superlayer phase is inherited from the Sm-E phase. These insights offer valuable knowledge about the nature of the Sm-E phase and the correlation between layered crystals, highlighting unique aspects of the crystallization process in layered OSCs and its strong connection to smectic LC phases.

The phase sequence of the LC phases enables solvent-free coating of polycrystalline films by forming an Sm-A membrane on the substrate and cooling it to room temperature. The emergence of the Sm-E phase during coating effectively suppresses crack formation, likely by mitigating the marked lattice change that can damage polycrystalline grain structures. In addition, the solvent-free coating process enables the formation of the superlayer cocrystal phase in polycrystalline films, which cannot be achieved through conventional solution-based processes. This feature facilitates the fabrication of solvent-free OTFTs, which exhibit hole transport characteristics with mobilities of ~1 cm^2^ V^−1^ s^−1^.

Since our solvent-free coating process can be conducted below 120°C, we expect it to be applicable to various substrates, such as glass coated with conductive indium tin oxide or poly(ethylene terephthalate) films, whose conductivity or mechanical properties are adversely affected by thermal treatments above 150°C (*55*, *56*). Although further studies are needed to improve the long-term stability of the present polycrystalline films, we expect that the additional treatments, such as encapsulation, could enhance the stability of the films by suppressing molecular motion within the films (*57*). We also anticipate that further investigation into the rate of film degradation as a function of thermodynamic variables such as temperature and pressure would provide deeper insights into the kinetics of phase transitions in these materials and aid in identifying conditions that ensure the long-term stability of the superlayer cocrystal film. These findings suggest that the phase behaviors of alkylated molecules can be controlled through molecular mixing and that liquid crystalline OSCs provide a promising route for fabricating OTFTs without the use of hazardous solvents.

## MATERIALS AND METHODS

### Characterization of thermodynamic properties

OSC molecules of mono-C_8_-BTBT and di-C_8_-BTBT were synthesized and purified as described elsewhere (*8*). The mixtures of mono-C_8_-BTBT and di-C_8_-BTBT were prepared by mixing the solution of each compound (dissolved with the concentration of 10 g liter^−1^ in the chlorobenzene, which was purchased from Fujifilm Wako Pure Chemical Corp., Japan) and crystallized by evaporating the solvent. Thermal properties of the compounds were characterized by a power compensation differential scanning calorimeter (DSC8500; PerkinElmer, US) equipped with a refrigerated cooler (IntraCooler II; PerkinElmer, US) under a nitrogen atmosphere. The textures of LC phases were observed by optical microscopy (VHX-6000; Keyence, Japan) where the temperature was controlled by a heat stage (10002L; Linkam, UK). The LC cells for POM texture observations were prepared by inserting the compounds between untreated cover glasses. The POM observations were performed using VHX-6000 equipped with polarizers under crossed-Nicols setting. Powder XRD measurements and thin-film XRD measurements were conducted by using Rigaku SmartLab with Cu Kα radiation. The powder samples were sealed in the Lindemann glass capillary with a diameter of 0.5 mm. The temperature-controlled powder XRD measurements were performed using Rigaku SmartLab equipped with a heat stage.

### Thin-film fabrication and device characterization

The crystalline thin films were fabricated by a solvent-free coating method (the detailed procedure of the film coating process is described in Results) on the p^+^-doped silicon wafer covered with a 100-nm-thick thermally grown SiO_2_ layer. The coating speed of the blade was controlled by a stepping motor (SHOT-302GS; Sigma Koki Co. Ltd., Japan). The temperature of the substrate during the coating process was controlled by a proportional-integral-derivative–controlled heat stage, where the temperature of the substrate surface was calibrated by using an infrared thermometer. The morphology of the crystalline films was probed by AFM (VN-8010; Keyence, Japan). The thickness of the films was characterized by AFM and confocal laser microscopy (VK-X200; Keyence, Japan). The OTFTs were fabricated by pattern source and drain electrodes by thermal evaporation of Au through a metal mask. The thickness of the Au electrodes (25 nm thick) was monitored by a quartz microbalance. The untreated SiO_2_ layer was used as the gate dielectric for OTFTs. The carrier transport properties of the OTFTs were measured by a semiconductor device parameter analyzer (E5270B; Agilent Technology, US) under a nitrogen atmosphere at room temperature.
