# Supplementary material for: Melt-mixed superlayer cocrystal formation using symmetric and unsymmetric organic semiconductors
Source: Sci Adv. 2025 Apr 4;11(14):eadv1878. doi: 10.1126/sciadv.adv1878 (PMC11970452; doi:10.1126/sciadv.adv1878)
Supplement: Supplementary file 1 — Supplementary Text Figs. S1 to S17 Tables S1 to S3 [file sciadv.adv1878_sm.pdf]

Science Advances
AAAS

Supplementary Materials for

Melt-mixed superlayer cocrystal formation using symmetric and unsymmetric organic semiconductors

Kiyoshi Nikaido *et al.*

Corresponding author: Kiyoshi Nikaido, nikaido-kiyoshi464@g.ecc.u-tokyo.ac.jp;
Tatsuo Hasegawa, t-hasegawa@ap.t.u-tokyo.ac.jp

Sci. Adv. **11**, eadv1878 (2025)
DOI: 10.1126/sciadv.adv1878

This PDF file includes:

Supplementary Text
Figs. S1 to S17
Tables S1 to S3

Supplementary Text

On the reliability of simulation of powder X-ray diffraction

As stated in the main text, the intensity of 00*l* Bragg peak intensities in powder X-ray diffraction (XRD) were reproduced through simulations based on the electron density distribution along the *z*-axis (normal to the molecular layer) for the superlayer phase of x_{di} = 0.5. In these simulations, we fixed the molecular structure of *di*-C_8-BTBT and *mono*-C_8-BTBT, i.e. the interatomic bond lengths, bond angles, and the conformations were fixed. Under these conditions, two free parameters were optimized: the Gaussian disorder (σ), representing fluctuations in the electron density due to the structural disorder, and Δz, the interlayer distance of BTBT cores within the unit cell (fig. S3A). To find the best fit to the experimentally observed diffraction intensities, we minimized the sum of the squared residual χ^2 defined as follows:

$$\chi^2 = \sum_{l=2}^{5} \left(\log_{10} I_{00l}^{(\mathrm{sim})} - \log_{10} I_{00l}^{(\mathrm{exp})} \right)^2$$

where $I_{00l}^{(\mathrm{sim})}$ and $I_{00l}^{(\mathrm{exp})}$ represent the normalized simulated and experimentally obtained diffraction intensity of 00*l* Bragg peaks, respectively. In our simulations, both σ and Δz were varied in increments of 0.05 Å (fig. S3B).

Figure S3B shows the map of χ^2 as a function of σ and Δz. As shown in fig. S3C, the intensities of 002, 003, and 005 Bragg peaks are relatively insensitive to variations in Δz. In contrast, the intensity of the 004 peak is highly sensitive to Δz, resulting in a pronounced dependence of χ^2 on Δz as depicted in fig. S3D. The χ^2 map in fig. S3B indicates a global minimum at σ = 0.65 Å and Δz = 20.50 Å. Notably, the optimal Δz = 20.50 Å is slightly longer than the molecular length of *mono*-C_8-BTBT (≈ 20.3 Å).

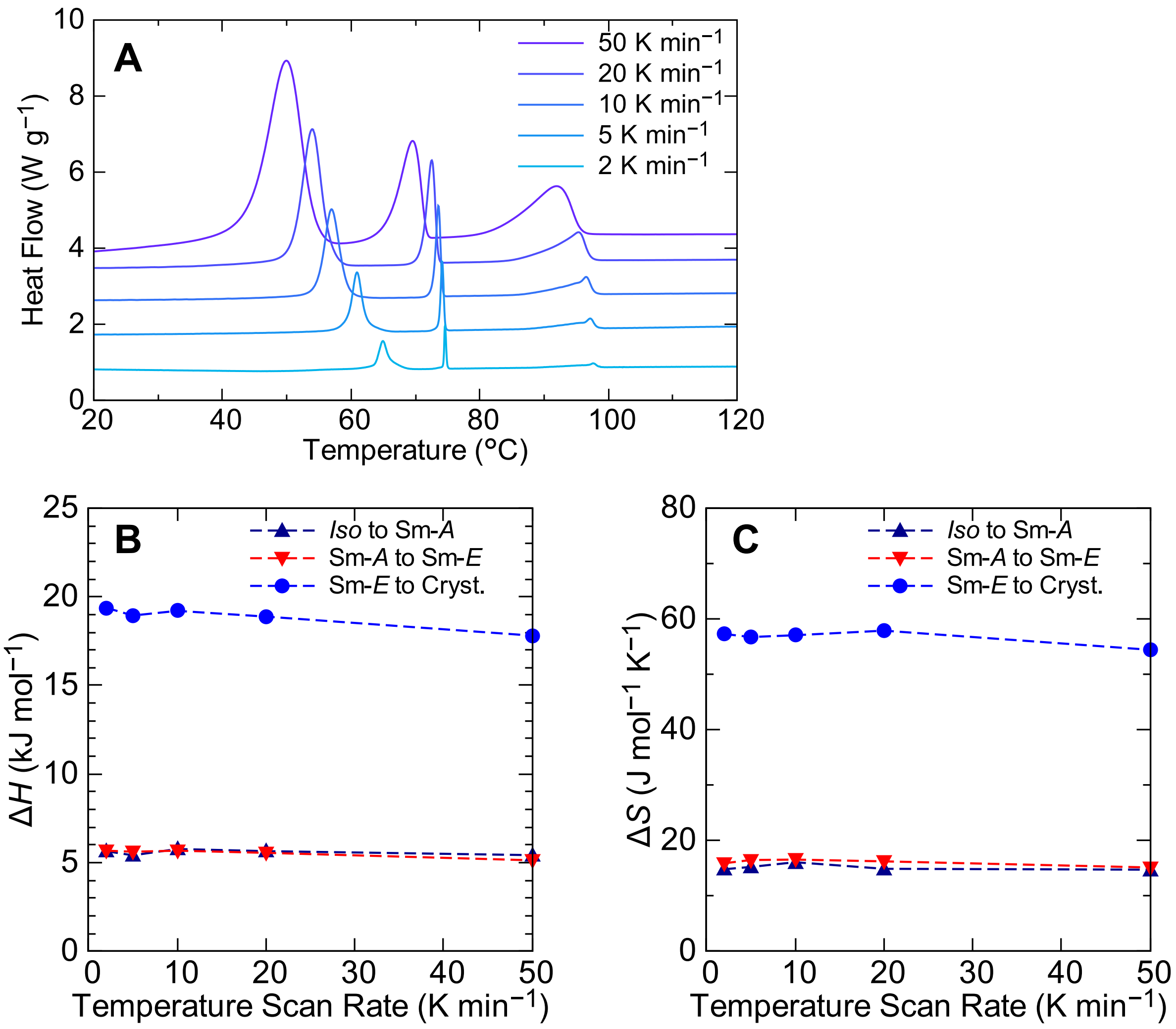

Fig. S1. DSC cooling scan for the equimolar mixture of *di*-C_8-BTBT and *mono*-C_8-BTBT (x_{di} = 0.5). (**A**) Cooling rate dependence of DSC charts. (**B**) Cooling rate dependence of the latent heat (ΔH) along with the phase transition. (**C**) Cooling rate dependence of the phase transition entropy (ΔS) along with the phase transition. The transition entropy was estimated by a procedure described elsewhere (*24*).

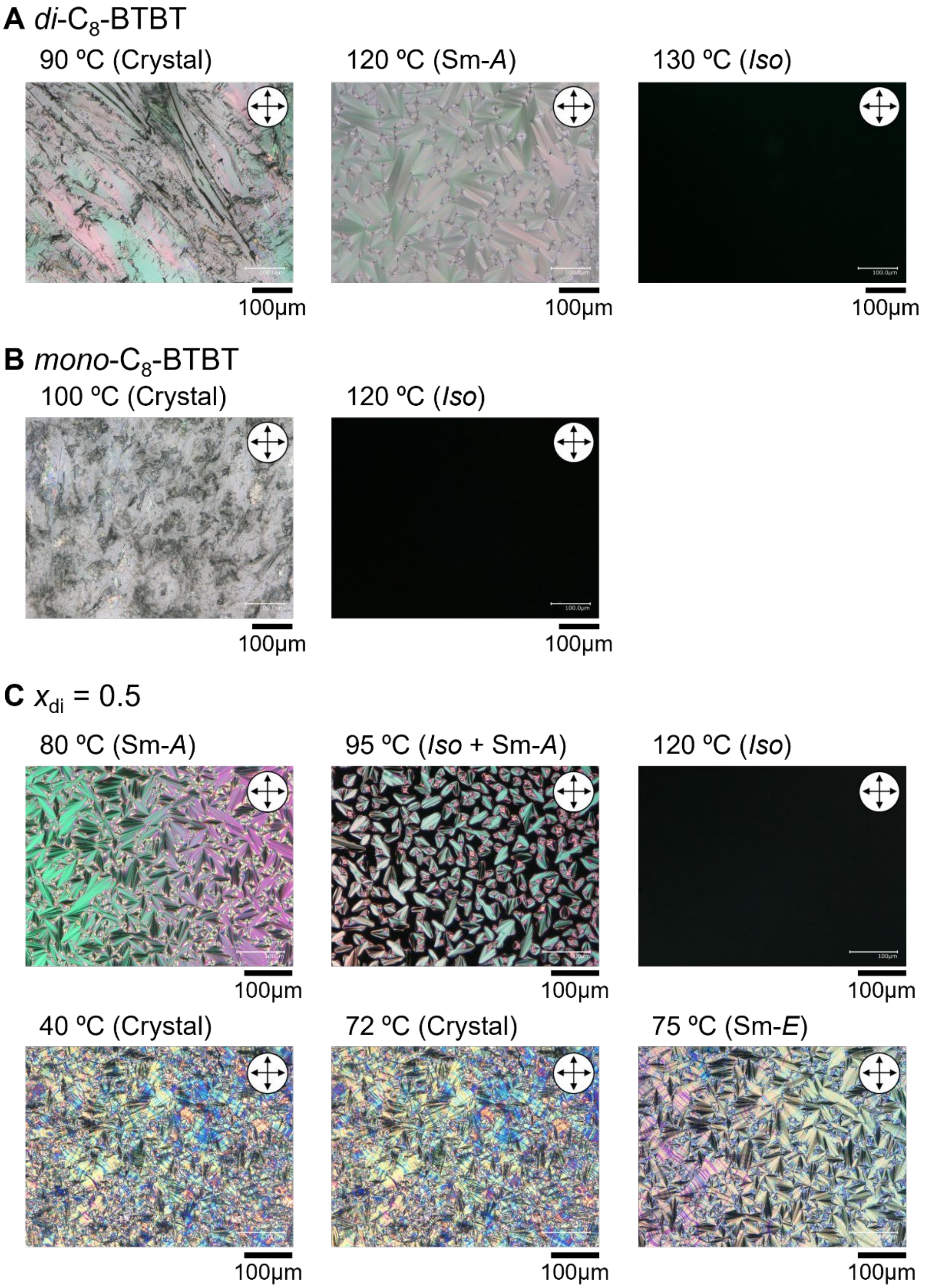

Fig. S2. POM textures of the pristine compounds. (**A**) *di*-C_8-BTBT and (**B**) *mono*-C_8-BTBT and (**C**) the equimolar mixture (x_{di} = 0.5) of *di*-C_8-BTBT and *mono*-C_8-BTBT during the cooling process.

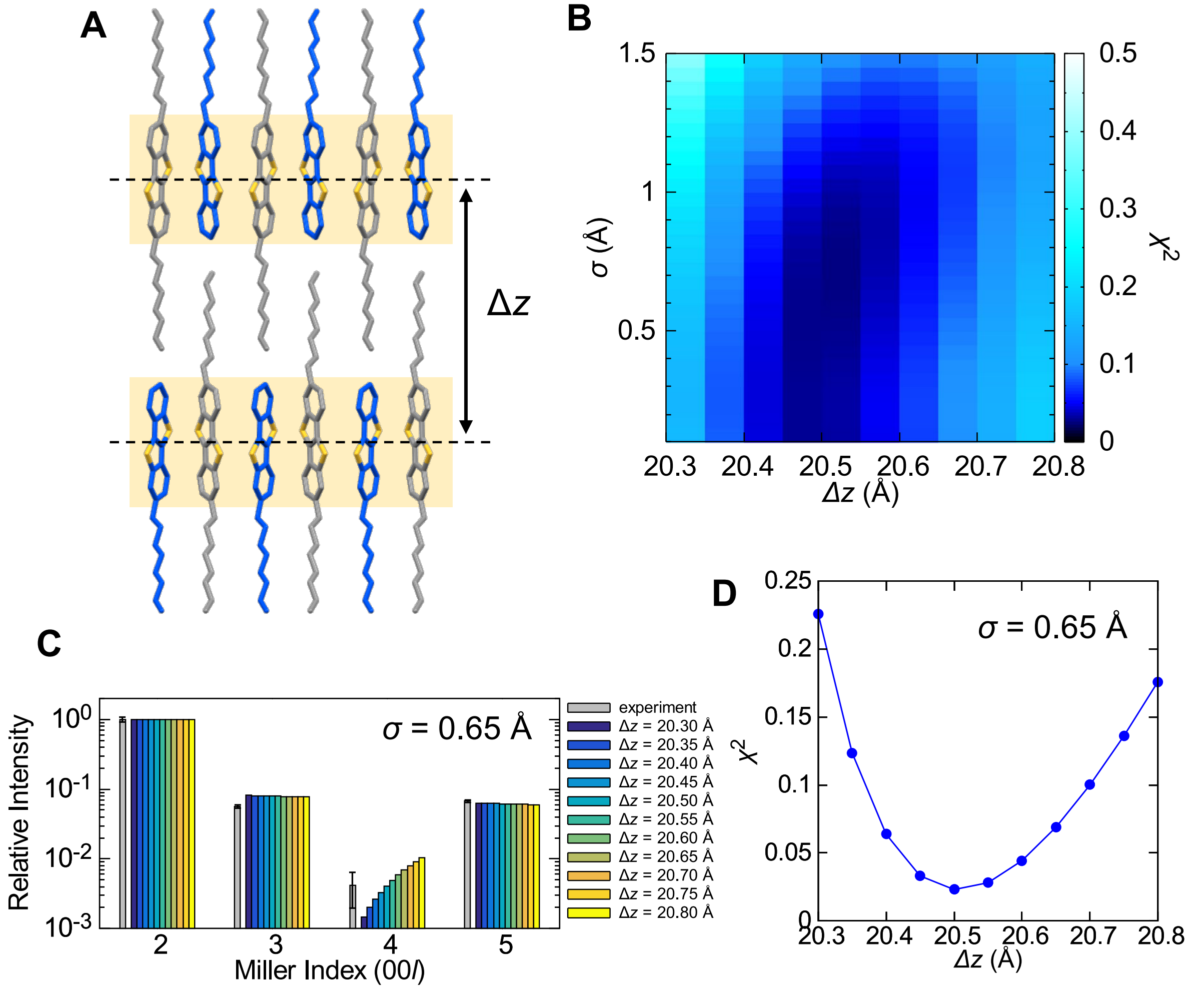

Fig. S3. Simulated intensity of (00*l*) Bragg diffraction in the powder XRD of x_{di} = 0.5 in the superlayer cocrystal phase. (**A**) Schematic of the layered structure of the superlayer phase of x_{di} = 0.5. The interlayer distance between the center of mass of BTBT core is defined as Δ*z*. (**B**) Map of the squared residual χ^2 depending on the Gaussian disorder σ and Δ*z*. (**C**) Δ*z*-dependence of simulated 00*l* intensities, where σ is fixed at 0.65 Å. The normalized intensity obtained from the powder-XRD experiment is also plotted, which is the same data as shown in Fig. 2C in the main text. (**D**) Δ*z*-dependence of χ^2 while σ is fixed at 0.65 Å.

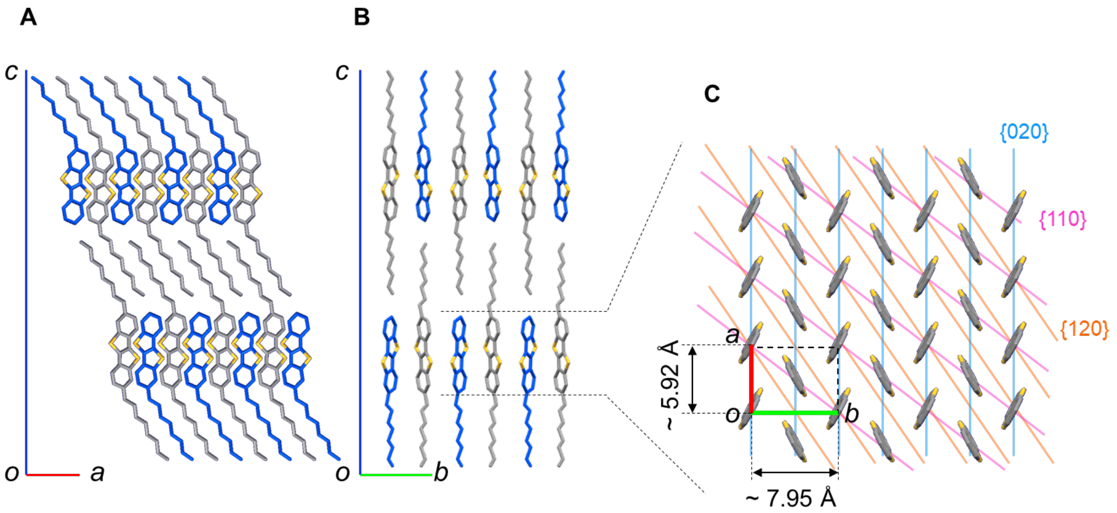

Fig. S4. Hypothetic structure of the superlayer cocrystal phase at x_{di} = 0.5. (**A**) Schematic of the structure of the superlayer cocrystal phase projected on (010) plane, assuming orthorhombic lattice. Molecules with blue and gray represent *mono*-C_8-BTBT and *di*-C_8-BTBT, respectively. (**B**) The schematic that projected on (100) plane. (**C**) Schematic for the in-plane herringbone arrangement of the π-conjugated cores, based on the lattice constant determined by the powder XRD measurements shown in Fig. 2A. Hydrogen atoms and the alkyl chains are omitted for clarity. The set of planes of {110}, {020} and {120} are schematically illustrated which exhibit primary diffraction peaks in the range of $q > 0.8$ Å^{-1} in Fig. 2A.

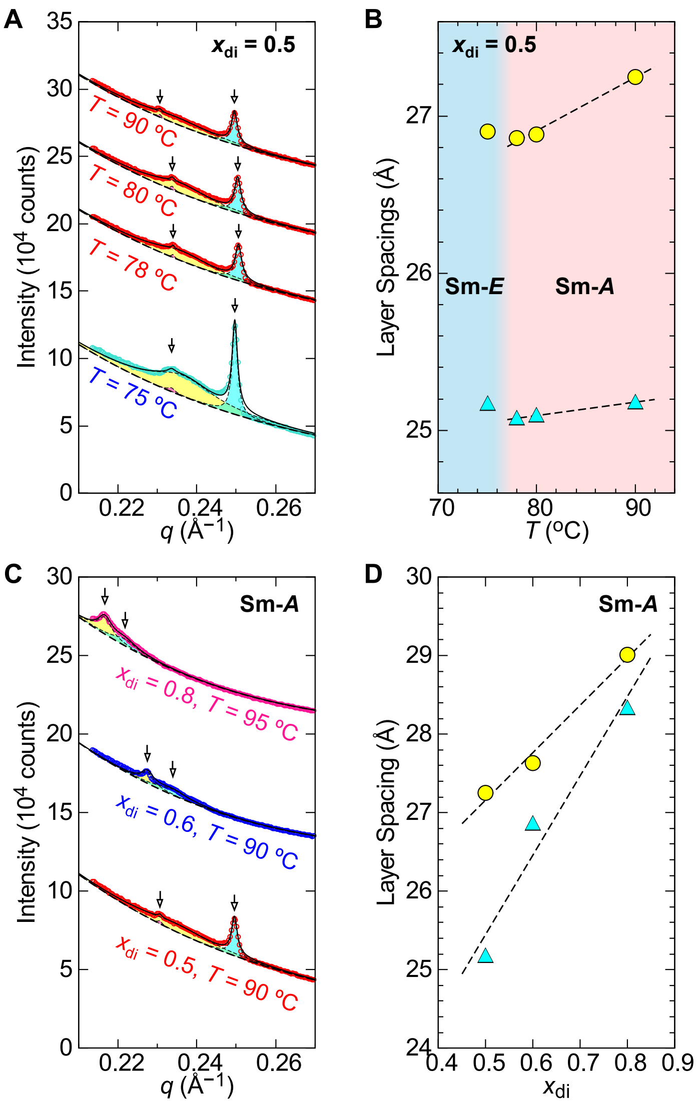

Fig. S5. Additional data on the temperature-controlled powder XRD diffractions of the mixtures of *di*-C$_8$-BTBT and *mono*-C$_8$-BTBT with various compositions. (**A**) Powder XRD profiles of x_{di} = 0.5 in LC phases. The 001 Bragg peaks are fitted by three Lorentzian functions, and the arrows indicate the positions of the primary Bragg peaks. The profiles are plotted with offset for clarity. (**B**) Temperature dependence of the layer spacings of x_{di} = 0.5. The dashed lines are plotted for eye-guide. (**C**) 001 Bragg peaks in Sm-*A* phase for x_{di} = 0.5, 0.6 and 0.8. The 001 Bragg peaks of x_{di} = 0.6 and 0.8 are fitted by two Lorentzian functions, and the peaks of x_{di} = 0.5 are fitted by three Lorentzian functions. The position of the primary Bragg peaks are indicated by arrows. The profiles are plotted with offset for clarity. (**D**) Composition dependence of the layer spacings in Sm-*A* phase. The dashed lines are plotted for eye-guide.

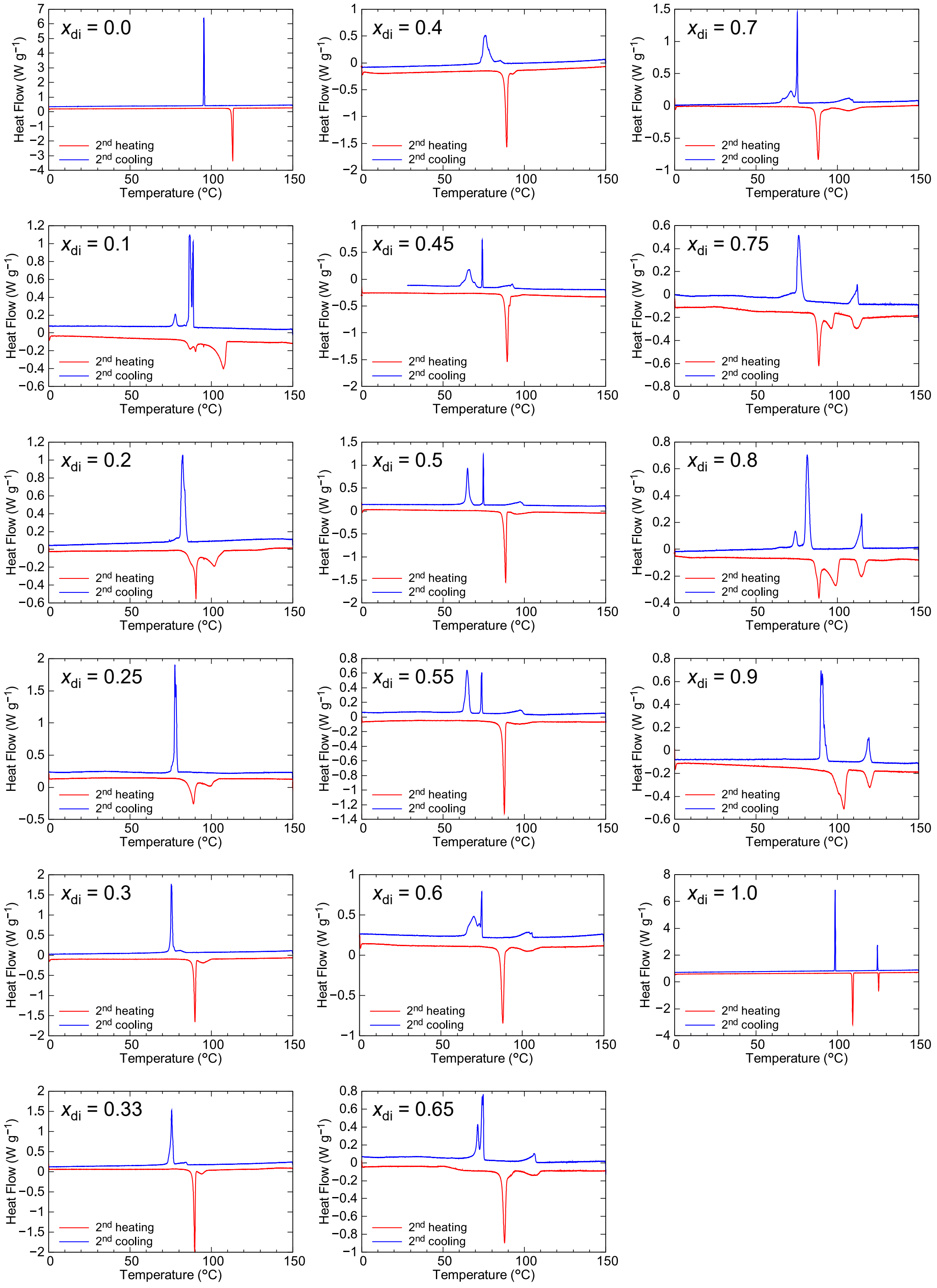

Fig. S6. DSC charts in the 2nd cycles of heating and cooling scans for the mixtures of *di*-C$_8$-BTBT and *mono*-C$_8$-BTBT with various compositions. Temperature scan rate is 2 K min^{-1}. The profiles are plotted with offset for clarity.

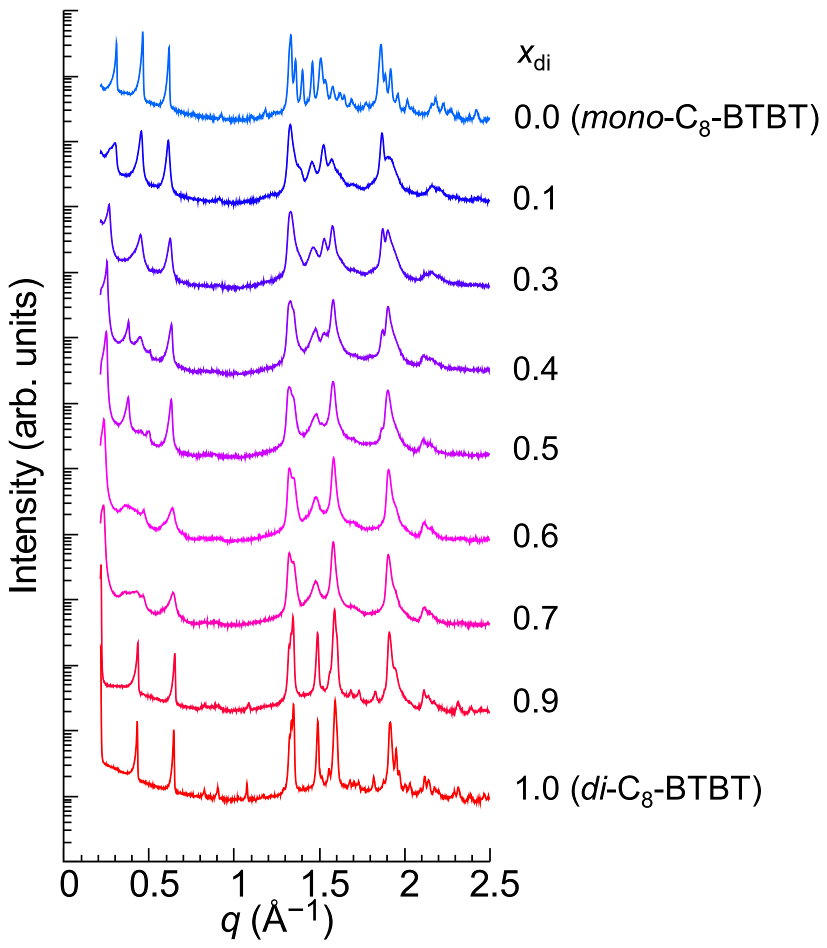

Fig. S7. Powder XRD profiles of the mixtures of *di*-C_8-BTBT and *mono*-C_8-BTBT with various compositions. The mixtures were fabricated by melt crystallization process. All the measurements were performed under ambient conditions.

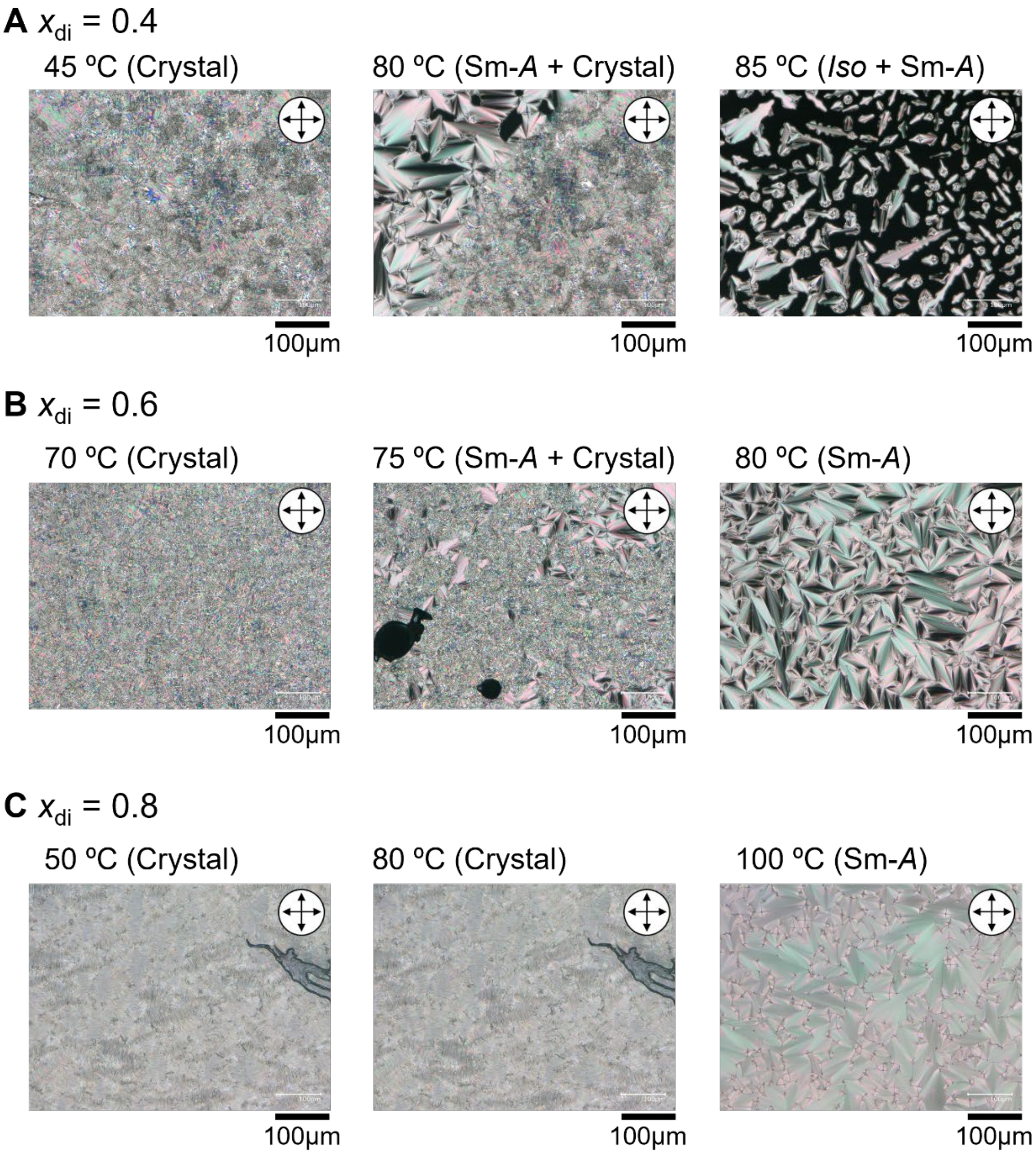

Fig. S8. POM textures of the mixtures of *di*-C_8-BTBT and *mono*-C_8-BTBT. (**A**) POM textures for the mixture with the composition of x_{di} = 0.4, (**B**) x_{di} = 0.6 and (**C**) x_{di} = 0.8 during the cooling process.

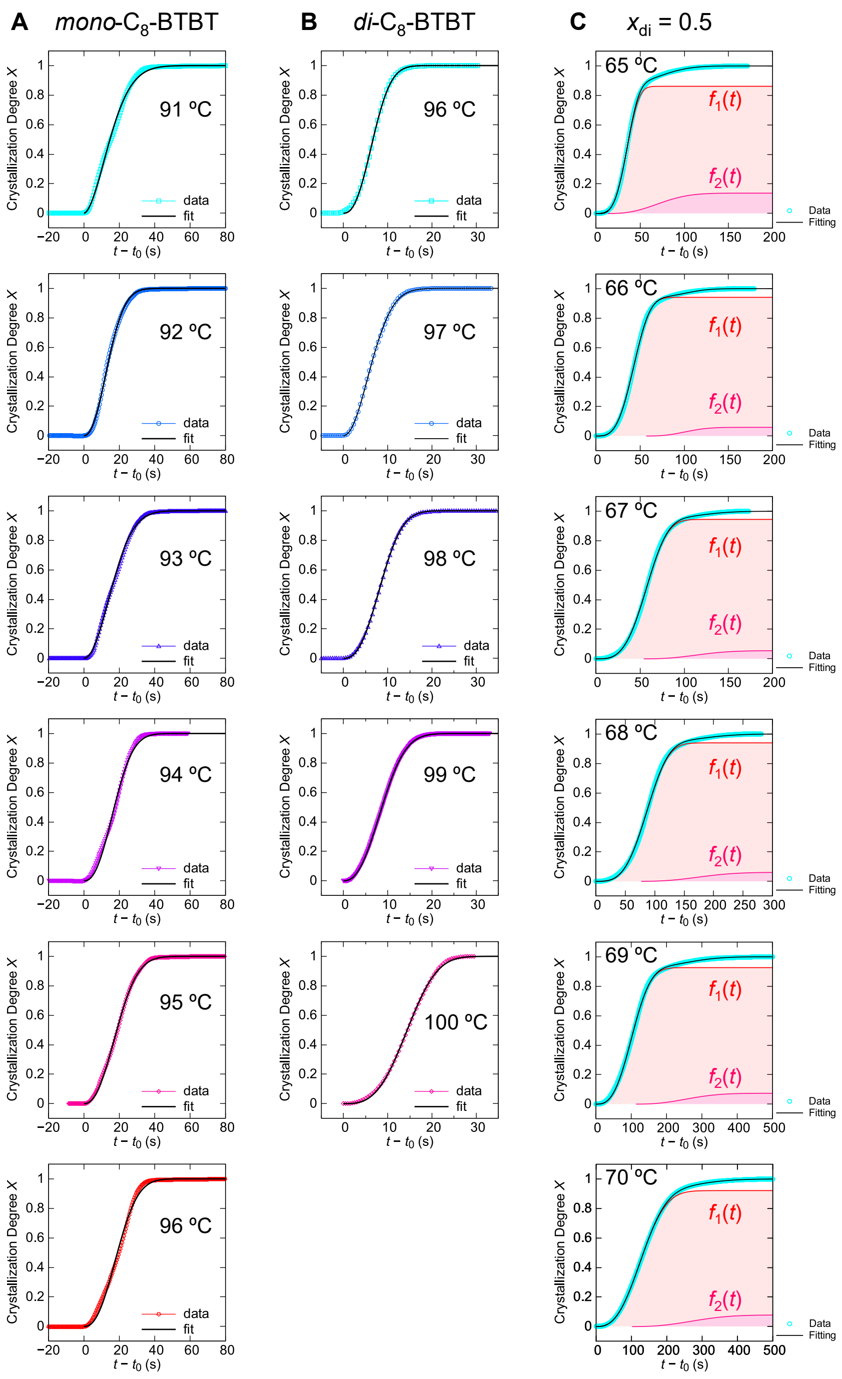

Fig. S9. Time evolution of the crystallization degree at fixed temperature. (**A**) *mono*-C_8-BTBT, (**B**) *di*-C_8-BTBT and (**C**) the equimolar mixture ($x_{\mathrm{di}} = 0.5$), along with the phase transition of *Iso* → Crystal, Sm-*A* → Crystal and Sm-*E* → Crystal, respectively. The black lines show the fitting curve by equation (4) in Figs. **A**, **B** and equation (5) in Fig. **C**. Here t_0 represents the incubation time which is the time required for nucleation to occur. All the plots were reproduced from the same data as shown in Fig. 5 in the main text.

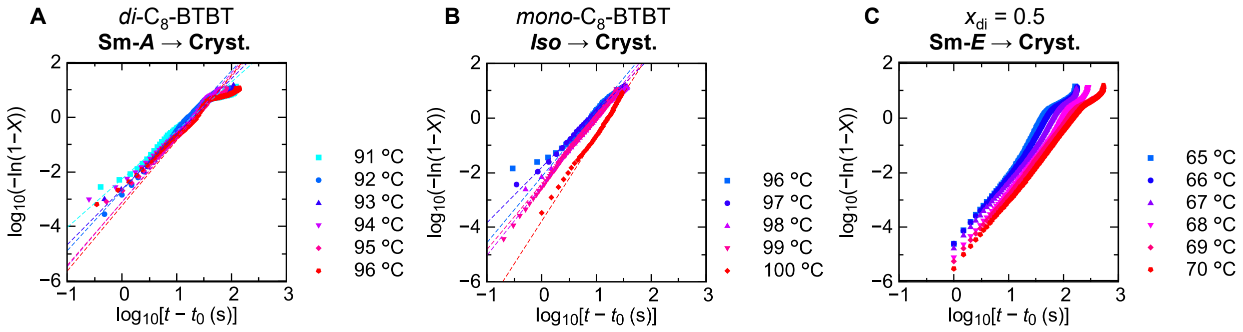

Fig. S10. Avrami plots for isothermal crystallization kinetics. (**A, B**) Avrami plots for the isothermal crystallization kinetics of *di*-C_8-BTBT and *mono*-C_8-BTBT, respectively. The dotted lines represent the fits using Equation (4). (**C**) Avrami plot for the isothermal crystallization of the mixture (x_{di} = 0.5). Since the crystallization kinetics of the mixture is described by Equation (5), the data deviate from a straight line in the Avrami plot.

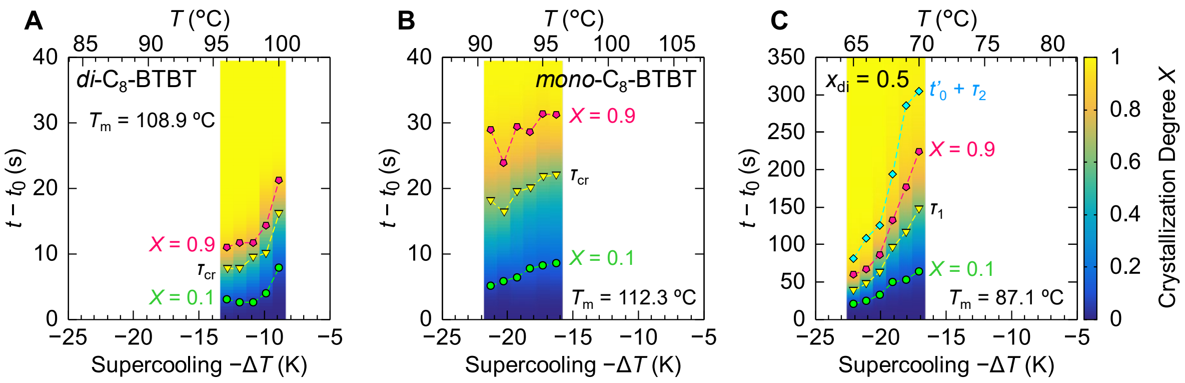

Fig. S11. Time-temperature-transformation (TTT) diagrams. (**A-C**) TTT diagrams for the melt-crystallization of *di*-C_8-BTBT, *mono*-C_8-BTBT and x_{di} = 0.5. The circles and pentagons in the Fig. represent the crystallization degree of X = 0.1 and 0.9, respectively. t_0 represents the incubation time and the supercooling temperature ΔT is defined as $T_m - T$, where T_m is the melting temperature.

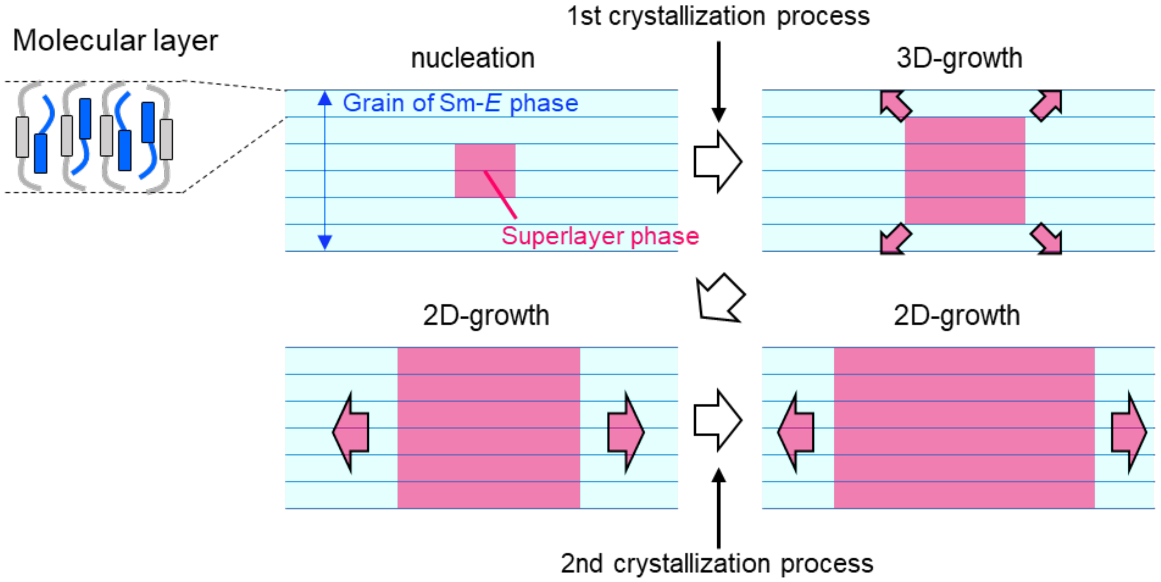

Fig. S12. Crystallization of the superlayer cocrystal phase. Schematics for the crystallization process in the equimolar mixture (x_{di} = 0.5) of *di*-C_8-BTBT and *mono*-C_8-BTBT during the phase transition from Sm-*E* to crystal phase.

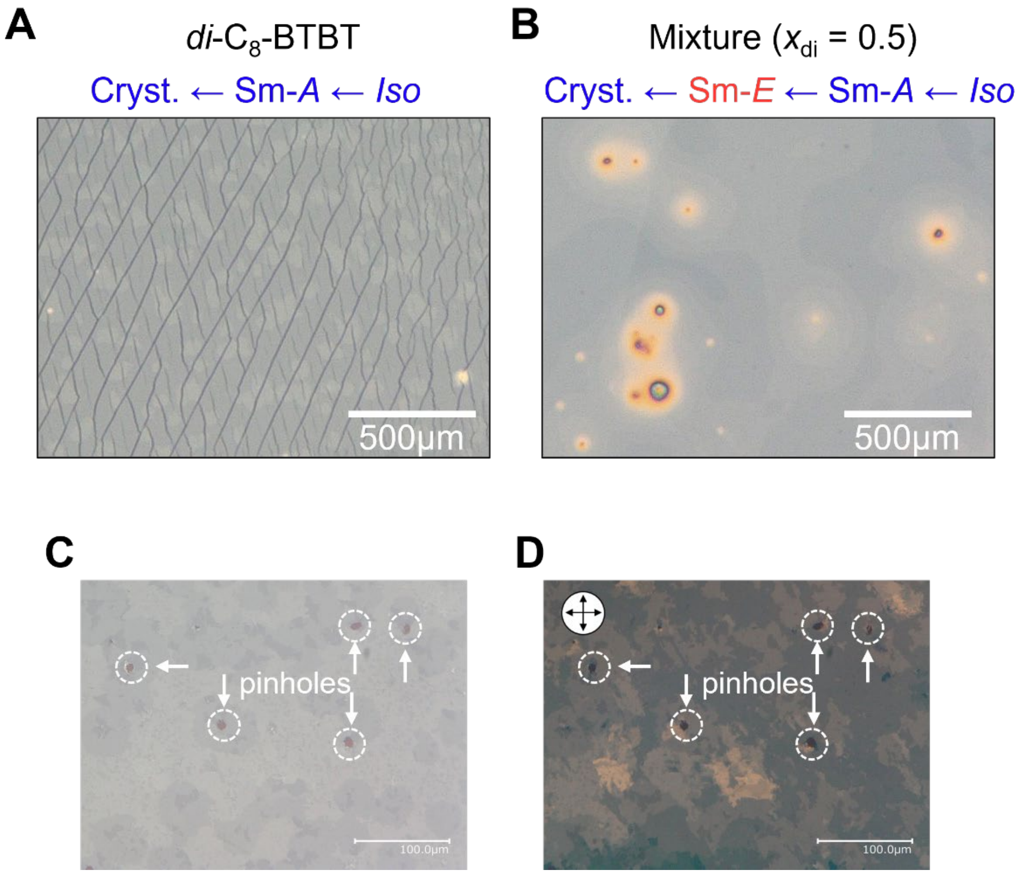

Fig. S13. Crystalline films obtained by the solvent-free coating procedure. (**A**) The optical microscope image of the crystalline film of the pristine compound of *di*-C_8-BTBT coated on Si/SiO_2 substrate. Here the compounds were melt at 150 ºC and then cooled down to T_{sub} = 131 ºC and the melt compounds were extended on the substrate with the blade-coating process with the coating speed of v = 5 μm s^{-1}, and finally cooled down to the room temperature. (**B**) The optical microscope image of the crystalline films of the equimolar mixture which was fabricated by the solvent-free coating procedure as described in the main text. Here the coating temperature (T_{sub}) and coating speed were set to T_{sub} = 98 ºC and v = 5 μm s^{-1}, respectively. (**C**, **D**) The magnified optical microscope image and POM image of the crystalline film of the equimolar mixture fabricated by the solvent-free coating. The pinholes are indicated by the dashed circles.

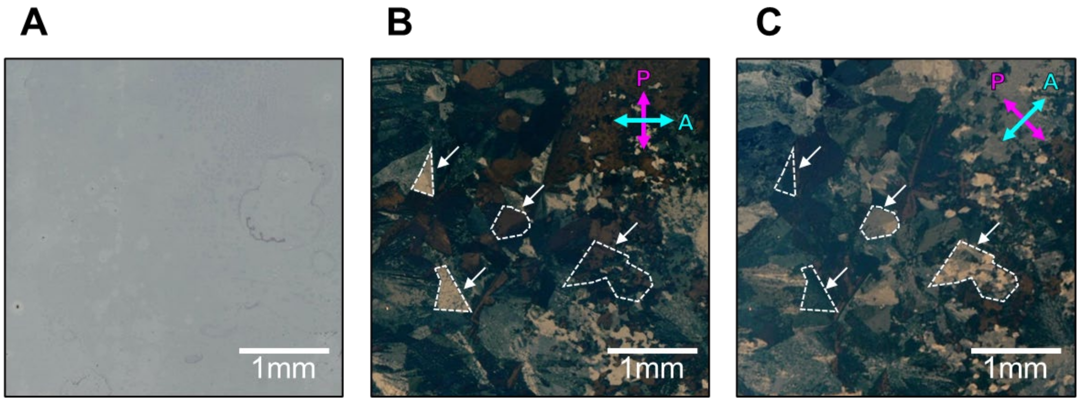

Fig. S14. POM observations of the solvent-free coated crystalline film of a mixture of *mono*-C_8-BTBT and *di*-C_8-BTBT (x_{di} = 0.5). (**A**) Optical microscope image of the crystalline film of the mixture (x_{di} = 0.5), fabricated using the solvent-free coating method at T_{sub} = 98 ºC. (**B**) POM image of the crystalline film with x_{di} = 0.5, showing the same area as in (A). The notations P and A in the figure indicate the directions of the polarizer and analyzer, respectively. (**C**) POM image of the same area as in (B), taken with the polarizer and analyzer rotated by 45º. The arrows and dotted-line-surrounded areas highlight grains where contrast variations due to orientation differences are visible.

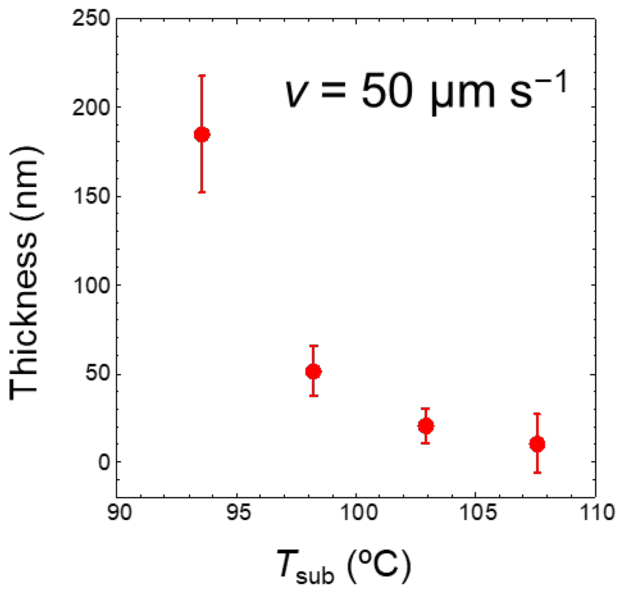

Fig. S15. Relation between substrate temperature and thickness of the solvent-free coated films. Experimentally determined relationship between thickness of the solvent-free coated crystalline films of the equimolar mixture and the coating temperature (T_{sub}) at a fixed coating speed (v = 50 μm s^{-1}).

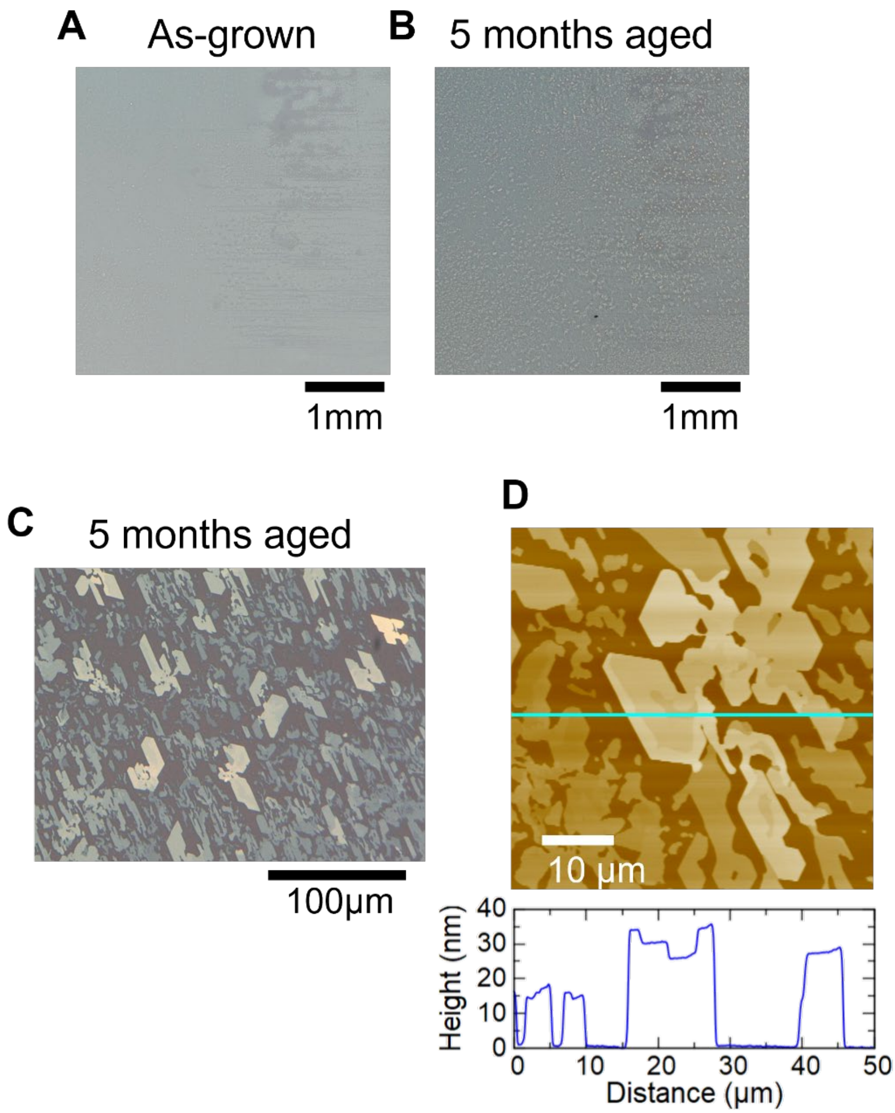

Fig. S16. Aging effect on the solvent-free coated film of the equimolar mixture. (**A**) Optical microscope image of the as-grown sample and (**B**) 5-months-aged sample. Aging was proceeded under ambient conditions. (**C**) Magnified image of the 5-months-aged film. (**D**) AFM topography of the 5-months-aged film.

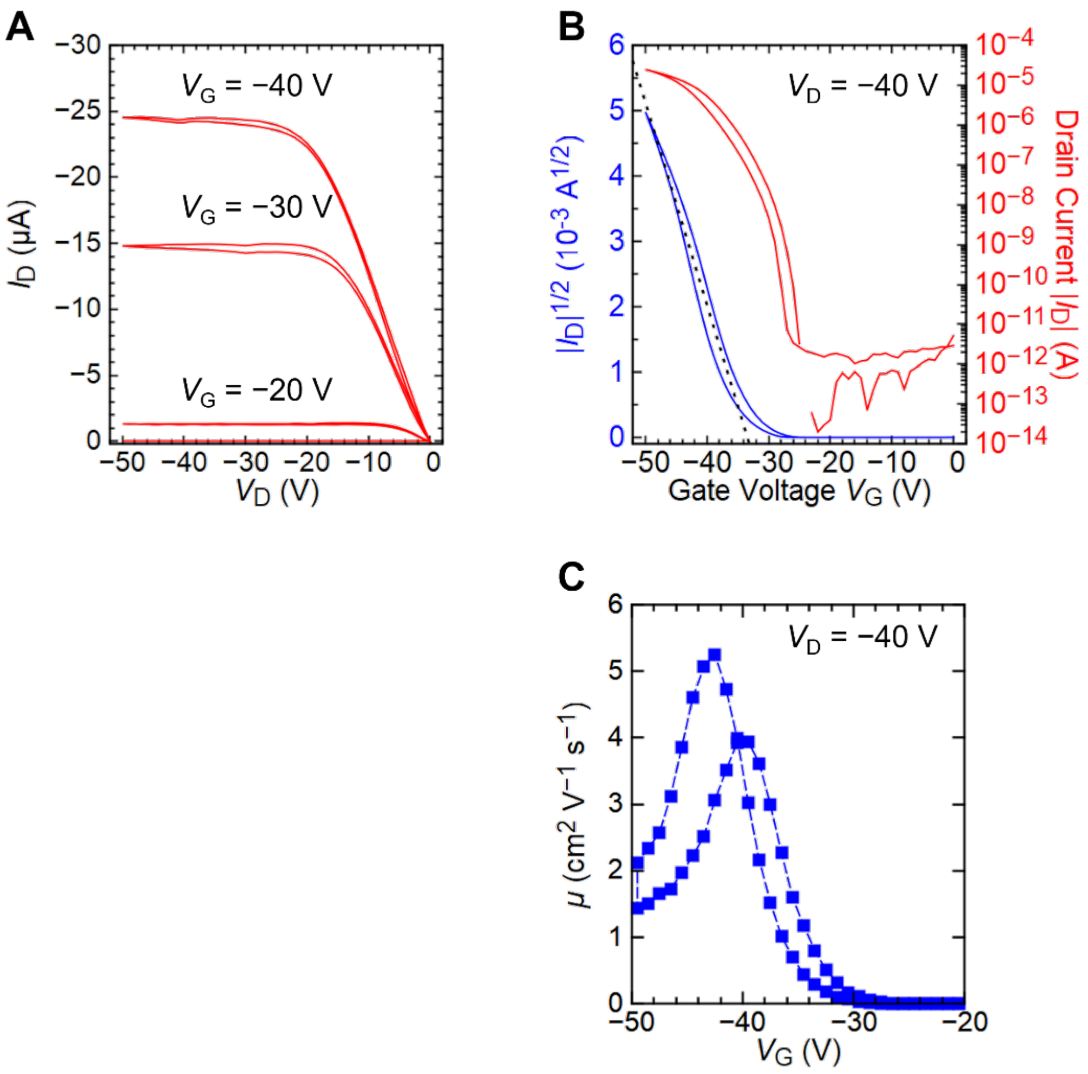

Fig. S17. OTFT characteristics of the solvent-free coated polycrystalline film of the equimolar mixture obtained within a day after the deposition of the Au electrodes as source and drain electrodes. (**A**) Output characteristics. (**B**) Transfer characteristics in the saturation regime. The channel length L and channel width w are L/w = 200 μm/415 μm which leads to the extracted mobility of 2.7 cm^2 V^{-1} s^{-1} by using the slope indicated by a dashed line. (**C**) Gate voltage dependence of the field-effect mobility.

Table S1. Optimized fitting parameters of Avrami exponents (*n*) and crystallization time (τ_{cr}) for *di*-C_8-BTBT. The fitting parameters were determined by fitting the time-evolution of isothermal crystallization degrees from Sm-*A* phase using equation (4) in the main texts.

Temperature	96 ºC	97 ºC	98 ºC	99 ºC	100 ºC
n	2.408(2)	2.025(9)	2.52(1)	2.396(3)	3.11(2)
τ_{cr} (s)	7.78(2)	7.76(1)	9.76(1)	10.123(4)	16.22(2)

Table S2. Optimized fitting parameters of Avrami exponents (*n*) and crystallization time (τ_{cr}) for *mono*-C_8-BTBT. The fitting parameters were determined by fitting the time-evolution of isothermal crystallization degrees from isotropic phase using equation (4) in the main texts.

Temperature	91 ºC	92 ºC	93 ºC	94 ºC	95 ºC	96 ºC
n	1.77(2)	2.19(2)	2.02(2)	2.35(4)	2.30(2)	2.39(4)
τ_{cr} (s)	18.11(8)	16.36(4)	19.52(5)	20.07(9)	21.83(5)	22.0(1)

Table S3. Optimized fitting parameters of Avrami exponents (*n*) and crystallization time (τ_{cr}) for the mixture of x_{di} = 0.5. The fitting parameters were determined by fitting the time-evolution of isothermal crystallization degrees from Sm-*E* phase using equation (5) in the main texts.

Temperature	65 ºC	66 ºC	67 ºC	68 ºC	69 ºC	70 ºC
n_1	3.69(2)	3.35(2)	3.43(1)	3.40(1)	2.88(1)	2.712(2)
τ_1 (s)	38.76(6)	47.73(6)	63.4(1)	96.0(2)	116.4(1)	147.2(2)
n_2	2.56(1)	2.66(1)	2.51(4)	2.46(4)	2.46(1)	2.506(4)
τ_2 (s)	67(4)	51.7(6)	72(2)	117(3)	172(2)	203(3)
A	0.86(1)	0.941(1)	0.945(3)	0.939(3)	0.926(1)	0.921(2)
$t'_0 - t_0$ (s)	14(5)	57.1(6)	54(3)	77(5)	114(3)	102(5)
